# Supplementary material for: Structure and dynamics determine G protein coupling specificity at a class A GPCR
Source: Sci Adv. 2025 Mar 19;11(12):eadq3971. doi: 10.1126/sciadv.adq3971 (PMC12101717; doi:10.1126/sciadv.adq3971)
Supplement: 20250319-1 [file sciadv.adq3971.v1.pdf]

STRUCTURAL BIOLOGY

Structure and dynamics determine G protein coupling specificity at a class A GPCR

Marina Casiraghi[1]†, Haoqing Wang[1], Patrick C. Brennan[2], Chris Habrian[1], Harald Hübner[3,4], Maximilian F. Schmidt[3,4], Luis Maul[3,4], Biswaranjan Pani[5], Sherif M. F. M. Bahriz[6], Bing Xu[6,7], Nico Staffen[3,4], Tufa E. Assafa[8]‡§, Bohan Chen[9], Elizabeth White[1], Roger K. Sunahara[9], Asuka Inoue[10,11], Yang K. Xiang[6,7], Robert J. Lefkowitz[5,12,13], Ehud Y. Isacoff[14,15], Nathaniel Nucci[16], Peter Gmeiner[3,4]*, Michael T. Lerch[2]*, Brian K. Kobilka[1]*

Copyright © 2025 The Authors, some rights reserved; exclusive licensee American Association for the Advancement of Science. No claim to original U.S. Government Works. Distributed under a Creative Commons Attribution License 4.0 (CC BY).

G protein–coupled receptors (GPCRs) exhibit varying degrees of selectivity for different G protein isoforms. Despite the abundant structures of GPCR–G protein complexes, little is known about the mechanism of G protein coupling specificity. The β_2-adrenergic receptor is an example of GPCR with high selectivity for Gαs, the stimulatory G protein for adenylyl cyclase, and much weaker for the Gαi family of G proteins inhibiting adenylyl cyclase. By developing a Gαi-biased agonist (LM189), we provide structural and biophysical evidence supporting that distinct conformations at ICL2 and TM6 are required for coupling of the different G protein subtypes Gαs and Gαi. These results deepen our understanding of G protein specificity and bias and can accelerate the design of ligands that select for preferred signaling pathways.

INTRODUCTION

There are more than 800 members of the G protein–coupled receptor (GPCR) superfamily (*1*), yet they couple with varying efficacy to only four G protein subfamilies (Gαs, Gαi/o, Gαq/11, and Gα12/13) to activate distinct downstream signaling cascades (*2*). In recent years, the structures of more than 400 GPCR–G protein complexes with different G protein subtypes have been reported (*3*–*9*). However, the molecular determinants of GPCR–G protein coupling specificity remain largely unknown (*10*–*14*). In addition, mutagenesis and phylogenetic analysis have found no correlation between sequence and coupling selectivity (*15*, *16*). Biophysical investigations have shown that GPCRs are inherently flexible, existing in an equilibrium of multiple conformations (*17*–*20*). Depending on their efficacy, ligands can shift this equilibrium toward specific states, facilitating the coupling of signaling partners. However, partner-specific states are likely transient, low-probability conformations that cannot be trapped by structural methods such as x-ray crystallography or cryo–electron microscopy (cryo-EM). Therefore, additional biophysical studies are needed to complement the information provided by structures and to delineate the transient yet important conformational states stabilized in the absence of bound G proteins (*21*–*24*).

A deeper understanding of the mechanism at the basis of G protein specificity is essential for the development of drugs that preferentially activate a single G protein subtype. This could reduce the potential adverse effects associated with the activation of multiple G protein isoforms. In this context, the development of biased ligands that preferentially activate a single G protein subtype is highly desirable as a tool to better characterize GPCR signaling and for therapeutic purposes. However, our understanding of the molecular determinants underlying biased signaling is still fragmentary, suggesting the need for a more detailed description of the conformational states adopted by receptors bound to biased ligands (*21*–*24*).

We chose the β_2-adrenergic receptor (β_2AR) as a prototypical class A GPCR to investigate G protein specificity and biased signaling. β_2AR and β_1-adrenergic receptors (β_1AR) are GPCRs expressed in cardiac myocytes and play essential roles in the regulation of cardiac function by the sympathetic nervous system. β_1AR and β_2AR primarily couple to the stimulatory G protein for adenylyl cyclase Gαs, to increase heart rate and contractility (*25*). β_2AR also binds to the Gαi subtype, the inhibitory G protein for adenylyl cyclase; activation of Gαi by the β_2AR can counteract the effects of Gαs activation on heart rate and contractility (*26*). β_2AR signaling through Gαi can also lead to activation of MAPK/ERK and PI-3K pathways. Chronic stimulation of the Gαs pathway leads to pathologic changes in the heart including myocyte apoptosis, which ultimately leads to congestive heart failure. In contrast, β_2AR activation of Gαi has a cardioprotective effect by activating the PI3K-Akt signaling cascade (*27*, *28*). However, increased Gαi signaling by the β_2AR has also been linked to the acceleration of pathologic changes in nonischemic models of heart failure (*29*), underlying the importance of understanding the molecular basis of the promiscuous signaling through both Gαs and Gαi.

[1]Department of Molecular and Cellular Physiology, Stanford University School of Medicine, Stanford, CA, USA. [2]Department of Biophysics, Medical College of Wisconsin, Milwaukee, WI, USA. [3]Department of Chemistry and Pharmacy, Medicinal Chemistry, Friedrich-Alexander-Universität Erlangen-Nürnberg, Erlangen, Germany. [4]FAU NeW, Erlangen, Germany. [5]Department of Medicine, Duke University Medical Center, Durham, NC, USA. [6]Department of Pharmacology, University of California, Davis, Davis, CA, USA. [7]VA Northern California Health Care System, Mather, CA, USA. [8]Department of Chemistry and Biochemistry, University of California, Santa Cruz, Santa Cruz, CA, USA. [9]Department of Pharmacology, University of California San Diego School of Medicine, La Jolla, CA, USA. [10]Graduate School of Pharmaceutical Sciences, Tohoku University, 6-3, Aoba, Aramaki, Aoba-ku, Sendai, Miyagi 980-8578, Japan. [11]Graduate School of Pharmaceutical Sciences, Kyoto University, 46-29 Yoshida-Shimo-Adachi-cho, Sakyo-ku, Kyoto 606-8501, Japan. [12]Department of Biochemistry, Duke University Medical Center, Durham, NC, USA. [13]HHMI, Duke University Medical Center, Durham, NC, USA. [14]Department of Molecular and Cell Biology, University of California, Berkeley, Berkeley, CA, USA. [15]Helen Wills Neuroscience Institute, University of California, Berkeley, Berkeley, CA, USA. [16]Department of Physics and Astronomy and Department of Biological and Biomedical Biosciences, Rowan University, Glassboro, NJ, USA.
*Corresponding author. Email: peter.gmeiner@fau.de (P.G.); mlerch@mcw.edu (M.T.L.); kobilka@stanford.edu (B.K.K.)
†Present address: Department of Biosciences, Università degli Studi di Milano, Milano, Italy.
‡Present address: Department of Chemistry and Chemical Biology, Cornell University, Baker Laboratory, Ithaca, NY 14853, USA.
§Present address: National Biomedical Resource for Advanced ESR Spectroscopy, Cornell University, Baker Laboratory, Ithaca, NY 14853, USA.

Gαs recruitment to the β$_2$AR has been characterized by structural and spectroscopic methods, including NMR and DEER spectroscopy (*4*, *17*, *18*, *23*, *30*–*33*). In contrast, limited information is available regarding the binding of Gαi to the β$_2$AR. Receptor phosphorylation has been proposed to play a role in Gαi recruitment at the β$_2$AR (*34*). However, previous biochemical studies showed that in vitro receptor phosphorylation with protein kinase A (PKA) failed to enhance Gαi coupling to the receptor (*35*). In this study, in vitro receptor phosphorylation with PKA failed to enhance G protein recruitment to the β$_2$AR with all the Gαi protein subtypes tested (Gαi$_1$, Gαi$_2$, and Gαi$_3$). Moreover, previous investigations in neonatal cardiac myocytes showed a biphasic coupling of the β$_2$AR to Gs followed by Gi. In this experiments, Gi coupling was not affected by the PKA inhibitor PKI (*36*).

In this work, we identified a Gαi-biased agonist (LM189) for the β$_2$AR and investigated the mechanism for its signaling bias using a combination of structural and biophysical methods. We observed that relative to nonbiased agonists, LM189 stabilizes a distinct conformation in TM6 and increases the dynamics of ICL2, explaining the preferential Gαi bias mediated by LM189 at this receptor system.

RESULTS

Development of the Gαi-biased ligand LM189

Structures of GPCRs coupled to the inhibitory G protein Gi show a smaller outward movement of TM6 compared to Gs and Gq/11 complexes (*3*–*7*, *9*, *37*). This has been attributed to the smaller size of the C terminus of the α5 helix of Gαi compared to the bulkier α5 of the Gαs and Gαq subtypes (*9*, *13*). A smaller outward displacement of TM6 is also observed in the crystal structure of the β$_2$AR bound to the partial agonist salmeterol (*38*). To screen for ligands that could stabilize the β$_2$AR-Gi complex for structure determination, we compared the effect of salmeterol with other β$_2$AR agonists for the coupling to Gs and Gi using the GTPase-Glo Assay (*32*) (Fig. 1, A and B) (note that we will use Gs and Gi, instead of Gαs and Gαi, when referring to the heterotrimer). Salmeterol is a subtype-selective β$_2$AR partial agonist for Gs activation relative to epinephrine, the endogenous β$_2$AR hormone also known as adrenaline (Fig. 1, A and C). Unexpectedly, the GTPase (guanosine triphosphatase) assay showed that salmeterol is more efficacious than epinephrine for β$_2$AR coupling to Gi (Fig. 1B). Salmeterol is a long-acting β$_2$AR agonist (LABA) composed of a saligenin ethanolamine pharmacophore and an aryloxy alkyl tail (*38*, *39*) (Fig. 1C). The high subtype selectivity for β$_2$AR is mediated by binding of the salmeterol tail in the receptor extracellular vestibule (also known as the exosite) (*38*). Salbutamol, another β$_2$AR partial agonist that shares the same saligenin ring as salmeterol but lacks the aryloxy alkyl tail, did not display high efficacy for Gi (Fig. 1, A to C). This suggests that the increased Gαi efficacy of salmeterol is, in part, mediated by its tail (Fig. 1C).

To better understand the increased efficacy of salmeterol for the coupling of β$_2$AR to Gi, we sought to determine the cryo-EM structure of the complex. Despite our efforts, we could not obtain the structure of the salmeterol-bound β$_2$AR-Gi complex. We identified

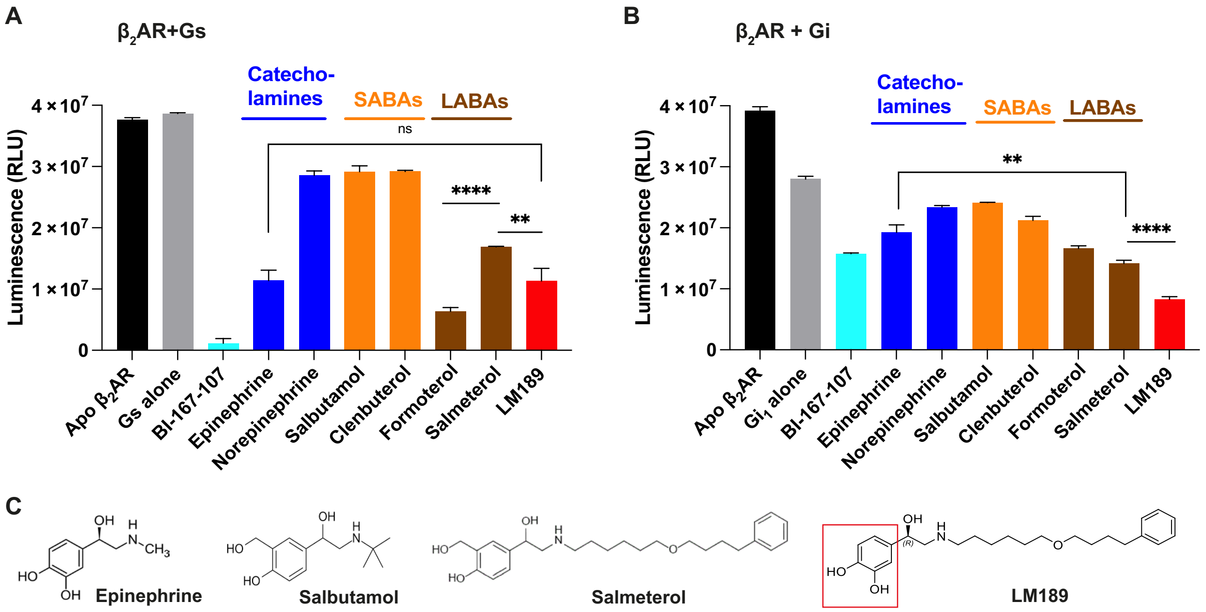

Fig. 1. Ligand efficacy at the β$_2$AR. (**A** and **B**) Luminescence GTPase-Glo assay. Ligand efficacy reflects the ability of the ligand-bound receptor to promote turnover of the G protein cycle. The reaction starts when β$_2$AR, bound to different ligands, is incubated with Gs (A) or Gi (B). At the end of the reaction, lower bars correspond to higher GTPase activity. (A) Salmeterol is a subtype-selective β$_2$AR partial agonist for Gs activation relative to other agonist such as epinephrine and formoterol (****$P < 0.0001$). LM189 is as efficacious as epinephrine at Gs turnover (ns, $P = 0.96$). In (B), salmeterol is more efficacious than epinephrine for β$_2$AR coupling to Gi (**$P = 0.003$). LM189 is more efficacious than epinephrine and salmeterol at coupling to Gi (****$P = 0.0001$). Experiments were performed as biological triplicates and results were plotted using GraphPad Prism. *P* values were calculated using the unpaired *t* test analysis on GraphPad Prism, assuming Gaussian distributions. ns = $P > 0.05$, *$P \leq 0.05$, **$P \leq 0.01$, ***$P \leq 0.001$, ****$P \leq 0.0001$. Data are represented as the mean ± SD. (**C**) Structures of β$_2$AR ligands. From the left, epinephrine (adrenaline) is the endogenous catecholamine neurotransmitter. Salbutamol is a partial agonist belonging to the short-acting β$_2$AR agonists (SABAs). Salmeterol is a long-acting β$_2$AR partial agonist (LABA), which exhibits a long duration of action and is used in the chronic management of asthma. Salmeterol has the same saligenin head group as salbutamol. LM189 was developed by replacing the saligenin moiety of salmeterol with a catechol group (red square).

conditions that led to a biochemically stable interaction; however, the complex dissociated upon sample vitrification. To further enhance the ligand efficacy for Gαi activation, we designed alternative ligands starting from the salmeterol scaffold. One of the ligands tested, named LM189, proved to be more efficacious than salmeterol at Gi turnover (Fig. 1, B and C). LM189 shares the tail region of salmeterol, while the saligenin moiety has been replaced by the catechol group, similar to epinephrine (Fig. 1C). LM189 is more efficacious than epinephrine and salmeterol at coupling to Gi (Fig. 1B) and as efficacious as epinephrine at Gs turnover (Fig. 1A). LM189 is equally efficacious for the Gi_1, Gi_2, and Gi_3 subtypes (fig. S1, A and B). For further experiments, we decided to focus on the Gi_1 subtype that will henceforth be referred to as Gi. Similar results in the GTPase assay were obtained with receptor reconstituted in high-density lipoproteins (HDLs; fig. S1C).

To quantify the degree of bias of LM189, we performed nano-bioluminescence resonance energy transfer (BRET) experiments between β_2AR-Rluc and mini-Gs-venus and mini-Gs/i-venus (fig. S1D). We obtained dose-response curves for Gs and Gi activation in the presence of epinephrine, the endogenous β_2AR ligand that we chose as reference, formoterol and LM189. We used the Operational Model equation (*40*, *41*) in GraphPad Prism to fit the data and determine the Log*R* values [equivalent to log(*t*/KA) ratios] (fig. S1D). We then calculated ΔLog(τ/KA) ratios, SEM, and relative effectiveness considering epinephrine as the reference ligand (*40*, *41*) (fig. S1D). ΔΔLog(τ/KA) ratios and bias factors (BFs) for LM189 activation of Gi were also determined. On the basis of our calculations, formoterol is weakly biased at Gi (BF = 3.7, fig. S1D), while LM189 shows notable bias toward Gi (BF = 24, fig. S1D).

We further characterized LM189-mediated β-arrestin recruitment profile, which we found similar to epinephrine (fig. S1E). On the basis of radioligand binding measurements, LM189 shows very high affinity for the β_2AR (K_i = 0.063 nM) but can also bind to the β_1AR with lower affinity (K_i = 28 nM) (fig. S1F and table S1). Collectively, our experimental data support the higher efficacy of LM189 over balanced ligands at Gi recruitment to the β_2AR.

Structure of the LM189-bound β_2AR-Gi complex

To determine the binding mode of LM189 and the structural determinants of Gi coupling, we obtained the structure of the β_2AR-Gi complex by cryo-EM to a global resolution of 2.9 Å (Fig. 2, A and B, and figs. S2 and S3, A to F). The overall structure of the LM189-β_2AR-Gi complex is similar to the previously determined BI-167107–β_2AR-Gs crystal structure (*4*) (Fig. 2B). The position of the α5 helix in the β_2AR-Gi structure closely resembles the β_2AR-Gs complex, in contrast to other GPCR-Gi complexes such as the μOR-Gi (*3*–*5*) (Fig. 2C). Conversely, TM6 of the receptor in the β_2AR-Gi complex displays a larger outward movement, more similar to the one observed in the β_2AR-Gs structure rather than the smaller TM6 opening reported for other GPCR-Gi structures (*3*–*5*) (Fig. 2C). Similar to the β_2AR-Gs structure, Phe $139^{34.51}$ of β_2AR ICL2 inserts into the hydrophobic pocket of Gi, formed by Phe 191 and Leu 194 of the β1 strand, Phe 336, Thr 340, Ile 343, Ile 344 of the α5 C terminus (Fig. 2D). However, compared to the Gs structure, engagement of Gi to the β_2AR is mediated by far fewer contacts between the G protein α5 and ICL2, TM3, and TM5 of the receptor, accounting for the diminished efficacy and less stable interaction of the β_2AR with Gi (Fig. 2D).

Although overall very similar, the β_2AR-Gi structure is most divergent from the β_2AR-Gs complex at the intracellular surface, in particular at ICL2, TM6, and TM5 (Fig. 2E and fig. S3, A to F). To increase complexing efficiency, the β_2AR-Gi complex was obtained with the aid of the PAM cmpd-6FA, which helps to stabilize ICL2 in the α-helical active-state conformation (*42*, *43*) (Fig. 2E and fig. S3, B and D). Therefore, our ICL2 structure may differ from that in a complex formed in the absence of cmpd-6FA (discussed further below). We do observe slight differences in the ICL2 active-state conformation of β_2AR-Gi compared to β_2AR-Gs, in particular at residues Lys $140^{34.52}$, Tyr $141^{34.53}$, and Gln $142^{34.54}$ (Fig. 2E and fig. S3, B and D). TM6 of β_2AR coupled to Gi is very similar to the β_2AR-Gs structure, with an almost identical outward movement (Fig. 2E and fig. S3E). However, by monitoring TM6 helix rotation at residue Glu $268^{6.28}$, we observed a slightly smaller outward rotation in the β_2AR-Gi complex compared to Gs (Fig. 2E), resulting in a ~3-Å difference in the outward tilt of Glu $268^{6.28}$.

In the β_2AR-Gs structure, the cytoplasmic end of TM5 undergoes a two-turn elongation compared to the inactive state. In contrast, the intracellular end of the TM5 helix of β_2AR-Gi elongates only one turn (*4*, *44*) (Fig. 2E and fig. S3, C and F); the cryo-EM density map suggests that this region is highly flexible. This can be attributed to the weaker network of contacts established between TM5 and Gi, mostly consisting of the hydrogen bonds between Asp 341 (Gi), Gln $229^{5.58}$ and Lys $232^{5.61}$ (β_2AR) (Fig. 2D). These interactions are present in the β_2AR-Gs structure as well, together with three additional hydrogen bonds that help to stabilize the complex (*4*). The shorter TM5 helix prevents a steric clash with the connector loop of the α4-β6 strand of Gi, which moves 6 Å closer to the receptor compared to the corresponding loop in Gs (*3*) (Fig. 2E and fig. S3F). Thus, the different TM5 conformations might be important determinants of G protein selectivity and might play a role in the initial steps of G protein coupling.

LM189 restricts the conformational heterogeneity of the ligand binding pocket

LM189 shares the same tail region as salmeterol, making similar hydrophobic and van der Waals interactions within the β_2AR exosite (*38*). LM189 also shares the same catechol head group of epinephrine, which forms hydrogen bonds with Asp $113^{3.32}$, Asn $312^{7.39}$, Ser $203^{5.42}$, Ser $207^{5.46}$ and Asn $293^{6.55}$ (Fig. 3, A to C). This polar network has been shown to be important for ligand efficacy at the β_2AR, and mutations of these residues have an impact on G protein and β-arrestin recruitment (*38*). We conducted molecular dynamics (MD) simulations to investigate the role of these polar interactions in ligand efficacy and bias. Rotameric analysis of Ser $207^{5.46}$ and Asn $293^{6.55}$ showed that ligand-receptor interactions in the orthosteric pocket of LM189-bound β_2AR were less heterogeneous and more stable compared to epinephrine-bound β_2AR (Fig. 3D). Ser $207^{5.46}$ and Asn $293^{6.55}$ clearly adopted a favored conformation for LM189-coupled receptor, whereas a broader ensemble of rotamers was sampled in the presence of epinephrine (Fig. 3D). For LM189, a very stable hydrogen bond network was formed between the ligand and Ser $203^{5.42}$, Ser $207^{5.46}$, and Asn $293^{6.55}$ (Fig. 3E). Moreover, Asn $293^{6.55}$ also formed a stable network with Tyr $308^{7.35}$ and Ser $204^{5.43}$ (Fig. 3E). In contrast, epinephrine stabilized a weaker polar network, resulting in the loss of the Asn $293^{6.55}$ and Ser $203^{5.42}$ interaction with the meta-OH group during our simulations (Fig. 3E).

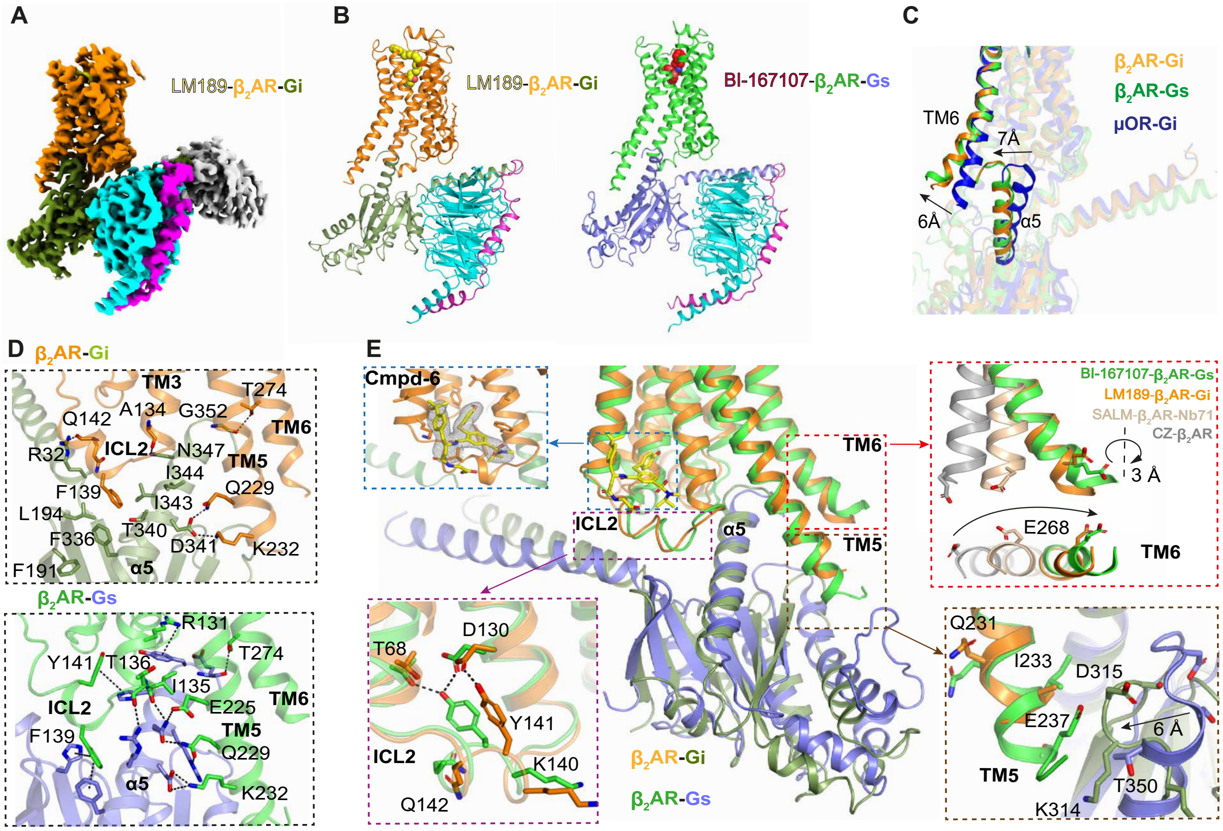

Fig. 2. Structure of the LM189-bound β_2AR-Gi complex. (**A**) Cryo-EM density map of the LM189-bound β_2AR-Gi-scfv16 complex. (**B**) Cryo-EM structure of LM189-bound β_2AR-Gi complex and crystal structure of BI-167107–bound β_2AR-Gs (*4*) (3SN6). β_2AR bound to Gi is colored orange, Gαi is in olive green, Gβ is in cyan, Gγ is in magenta, and scfv-16 is in gray. β_2AR bound to Gs is colored green, and Gαs is in slate. (**C**) Relative orientation of receptor TM6 and α5 helix of the β_2AR-Gi (orange), β_2AR-Gs (green), and μOR-Gi (blue) (*3*) complex structures. (**D**) Top: α5 engagement of Gi and the interactions formed with the ICL2, TM3, and TM5 of β_2AR. β_2AR is colored orange, and Gαi is in olive green. Bottom: α5 engagement of Gs and the interactions formed with the ICL2, TM3, and TM5 of β_2AR. β_2AR is colored green, and Gαs is in slate. Polar contacts within 4 Å are highlighted. (**E**) Structural differences between the LM189-bound β_2AR-Gi complex and BI-167107–bound β_2AR-Gs (*4*). Top left blue panel: The PAM cmpd-6FA (*42*, *43*) (yellow), binding at the top of ICL2. Lower left magenta panel: When bound to Gi, ICL2 of β_2AR adopts a slightly different conformation compared to Gs. Top right red panel: By monitoring TM6 rotation at Glu^{268}, the TM6 helix is slightly less rotated in the β_2AR-Gi structure compared to β_2AR-Gs (~3 Å). Carazolol-bound inactive-state β_2AR is colored gray (2RH1) (*44*), salmeterol-bound β_2AR in complex with Nb71 is colored wheat (6MXT) (*38*), LM189-bound β_2AR-Gi complex is colored orange, and BI-167107–bound β_2AR-Gs (3SN6) (*4*) is colored green. Lower right brown panel: TM5 of the β_2AR-Gi complex is one helix turn shorter than TM5 of the β_2AR-Gs structure, avoiding a steric clash between the tip of TM5 and the connecting loop between the a4 helix and b6 strand of Gi.

Additional MD simulations at the extracellular region of β_2AR bound to the ligands epinephrine, LM189, and salmeterol indicated a more flexible ECL3 in the epinephrine-bound state coupled to Gs (red box in fig. S3G) compared to LM189-coupled receptor, which displayed similar ECL3 motions to salmeterol-bound β_2AR (fig. S3G). In addition, we observed higher flexibility of ECL1 and ECL2 when β_2AR was coupled to Gi (fig. S3G). Collectively, our MD simulations suggest that LM189 restricts the conformational heterogeneity of the ligand-binding pocket compared to the more flexible configuration in the presence of epinephrine.

Because the β_2AR-Gi and β_2AR-Gs structures mostly diverge at ICL2, we conducted MD simulations at the intracellular cavity of the receptor to investigate ICL2 conformational changes in Gs- and Gi-bound receptors. Simulations of the LM189-β_2AR-Gi and epinephrine-β_2AR-Gs complexes after removal of the respective G proteins showed partial unwinding of the ICL2 helical structure (Fig. 3, F and G). We observed greater ICL2 mobility when the receptor was bound to LM189, as shown by the repositioning of residue Tyr 141$^{\text{ICL2}}$ (Fig. 3, F and G). This suggests increased ICL2 dynamics in the absence of cmpd-6FA and G proteins. Because MD simulation timescales are too short (nanoseconds to microseconds) to fully characterize the conformational changes occurring at the intracellular cavity (milliseconds), we decided to conduct additional biophysical measurements to better describe the intracellular rearrangements implicated in G protein specificity.

The role of ICL2 in G protein specificity

Hydrogen deuterium exchange mass spectrometry studies indicate that ICL2 interaction with the hydrophobic core of the G protein α subunit is one of the first steps in the receptor–G protein interaction (*13*, *31*, *33*). Previous biophysical and structural investigations have shown that ICL2 of β_2AR forms a loop in the inactive state (*44*) and a helix in the active-state conformation (*4*). In our β_2AR-Gi complex, ICL2 forms a partial helix (Fig. 2, D and E). However, as noted above, this might be due to the presence of the cmpd-6FA PAM, which binds on top of ICL2 and stabilizes the helical

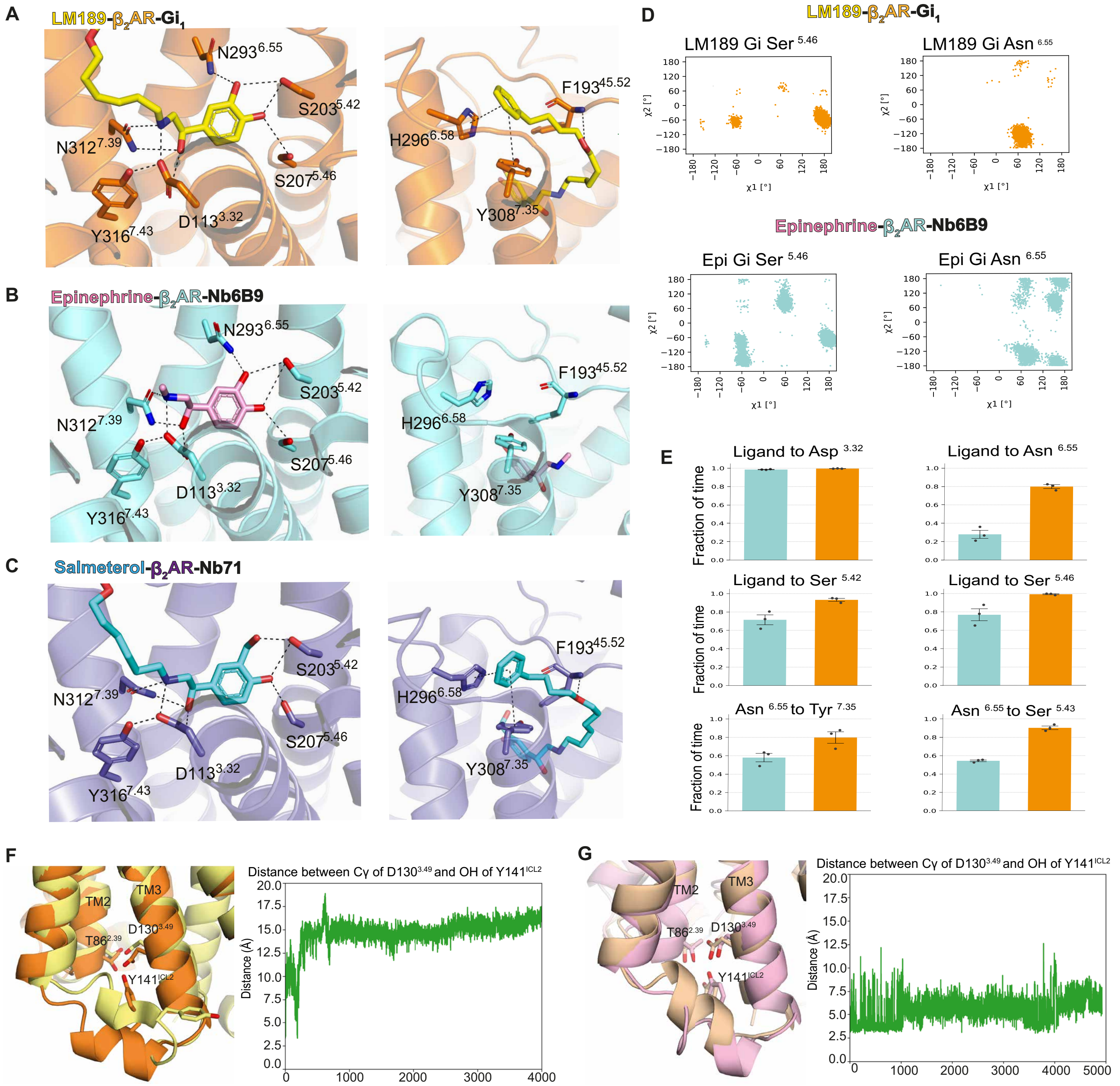

Fig. 3. Ligand-binding pocket of the LM189-bound β_2AR-Gi complex. (**A** to **C**) The orthosteric pocket (left) and exosite (right) of β_2AR bound to LM189 (A), epinephrine (B), and salmeterol (C). (A) LM189 is colored yellow, and β_2AR in the β_2AR-Gi structure is colored orange. (B) Epinephrine is colored pink, and epinephrine-bound β_2AR in complex with Nb80 (4LDO) (*67*) is colored aquamarine. (C) Salmeterol is colored blue, and salmeterol-bound β_2AR in complex with Nb71 (6MXT) (*38*) is colored slate. H-bonds are showed as dashed lines. (**D** and **E**) Molecular dynamics simulations of active-state β_2AR-Gi bound to LM189 or epinephrine. (D) Rotamer analysis of $Ser^{5.46}$ and $Asn^{6.55}$ of LM189-bound (orange, top) and epinephrine-bound (aquamarine, bottom) β_2AR-Gi. (E) Histograms represent hydrogen-bond formation frequencies as a fraction of time in three 2-μs MD simulations. β_2AR-Gi is represented in orange, and epinephrine-bound β_2AR-Gi is in aquamarine. (**F** and **G**) MD simulations at the intracellular cavity of β_2AR. (F) Left: Comparison of the cryo-EM structure of the LM189-bound β_2AR (orange) and a representative MD snapshot of the LM189-bound β_2AR (yellow). Right: Plot shows the progression of the distance between the Cγ of $Asp130^{3.49}$ and OH of $Tyr141^{ICL2}$ over the course of 4 μs. (G) Left: Comparison of the epinephrine-bound β_2AR model (pink, based on the BI-167107–bound β_2AR crystal structure, PDB: 3SN6) and a representative MD snapshot of the epi-bound β_2AR model (wheat). Right: Plot shows the progression of the distance between the Cγ of $Asp130^{3.49}$ and OH of $Tyr141^{ICL2}$ over the course of 4.9 μs.

conformation (*42*, *43*). Previous NMR studies suggest that ICL2 does not form a helix when coupled to Gi (*14*). We used continuous wave–electron paramagnetic resonance (CW-EPR) spectroscopy to investigate ICL2 conformational dynamics, to better understand its implication for G protein selectivity at the β_2AR. The EPR spectral line shape is sensitive to protein motion on the nanosecond timescale. For properly placed labels, conformational exchange that takes place on the microsecond or longer timescale results in a composite line shape composed of the weighted sum of spectral components arising from different conformational states (*45*). We monitored the conformational dynamics at ICL2 by site-directed spin labeling of Q142C, a residue located in the middle of ICL2, which served as a conformational reporter (Fig. 4A).

We collected CW spectra for the unliganded (apo) receptor and for β_2AR bound to the biased ligand LM189, the full agonist BI-167107, and the partial agonist salmeterol (fig. S4A). The spectrum of the apo receptor is dominated by a component reflecting the high mobility of the spin label, with a minor component reflecting an immobilized state (Fig. 4B and fig. S4A). On the basis of prior structural evidence, the mobile and immobile components are taken to reflect the loop and helical states of ICL2, respectively. Thus, the CW spectrum of the apo receptor indicates the presence of an equilibrium between a loop and the helical state of ICL2 (Fig. 4B and fig. S4A). Ligand binding did not substantially change the CW spectral line shape compared to the apo receptor, except for the case of LM189, where we observed an increase in the population of the mobile spectral component (Fig. 4B and fig. S4, A and B), in agreement with our MD simulations (Fig. 3F). Upon G protein coupling, we observed a decrease in the mobile component population and a concomitant increase in the immobile component (Fig. 4, C and D, and fig. S4, A and D), a change consistent with ICL2 transition from loop to helix. This shift was greater for Gs than for Gi coupling (Fig. 4, C and D). Helix formation upon Gs coupling was more pronounced in the salmeterol and LM189 conditions (Fig. 4D and fig. S4, A, B, and D). This suggests similar conformational dynamics at ICL2 of the receptor bound to LM189 and salmeterol upon Gs coupling.

Together, these observations suggest that ICL2 of β_2AR adopts a stable helix conformation when bound to Gs, while being more dynamic when bound to ligands and Gi. F139 of ICL2 of β_2AR inserts within the G protein hydrophobic pocket formed by the αN/β1 hinge, β2/β3 loop, and α5 (Fig. 2D). The persistence of the unstructured ICL2 conformation upon Gi coupling, when compared with Gs coupling, correlates with the less stable interactions that we observe in the β_2AR-Gi structure (Fig. 2D). Because ICL2 participates in the initial G protein recognition and engagement with the receptor, together with the α5 and distal C terminus of the G protein, different ICL2 conformations might also be associated with structurally distinct mechanisms for primary versus secondary G protein

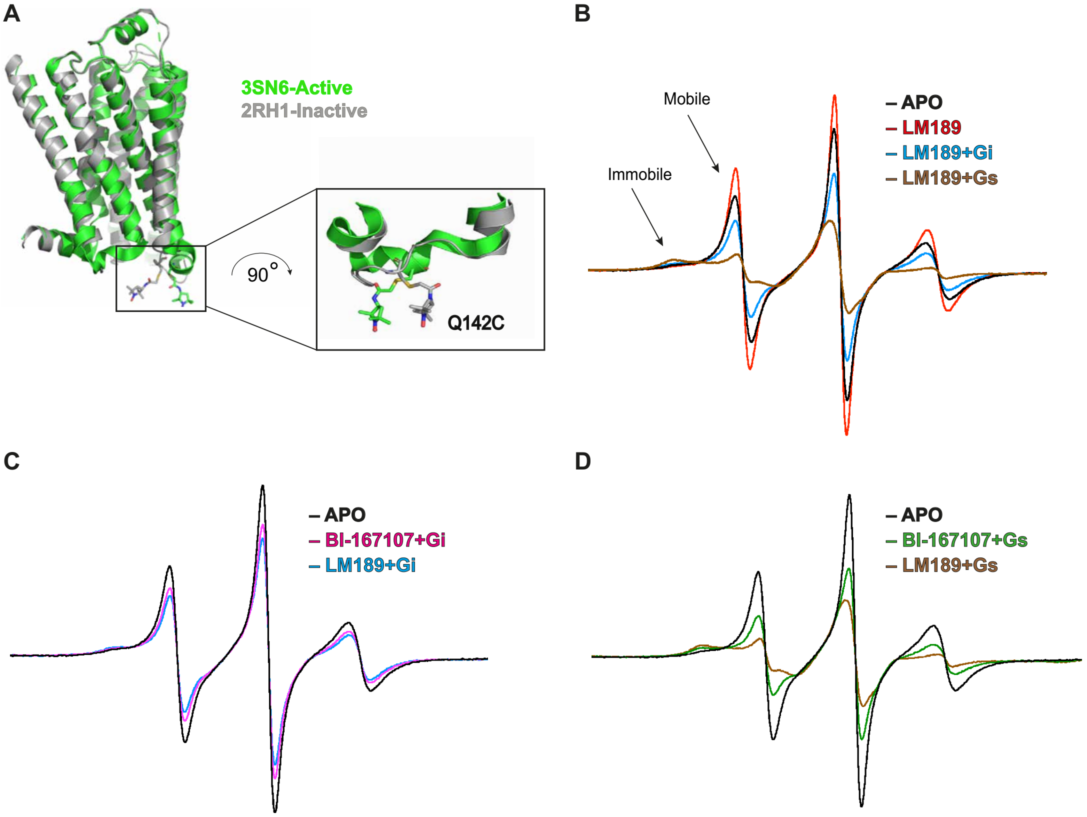

Fig. 4. CW-EPR studies of ICL2 of β_2AR. (**A**) A minimal-cysteine version of the β_2AR with an acetamido-PROXYL spin-label side chain shown at the mutated Q142C residue on ICL2 for EPR studies. Inactive-state β_2AR (2RH1) (*44*) is colored gray, and active-state β_2AR (3SN6) (*4*) is colored green. (**B**) Superimposed CW-EPR spectra of β_2AR in the apo, LM189-bound, and Gi- and Gs-protein bound conditions. The regions of the low-field line dominated by mobile and immobile components are indicated. (**C**) Superimposed CW-EPR spectra in the apo, LM189-bound, and BI-167107–bound receptor in complex with Gi. (**D**) Superimposed CW-EPR spectra in the apo, LM189-bound, and BI-167107–bound receptor in complex with Gs. All spectra are area normalized and color coded as indicated.

coupling. This agrees with previous NMR and mass spectrometry findings that propose a different role for ICL2 in G protein recruitment for Gs- and Gi-coupled receptors (*13*, *14*, *31*, *33*).

LM189 stabilizes a Gi-specific TM6 conformation

TM6 outward movement has been investigated with a variety of biophysical methods to monitor GPCR activation (*17*, *18*, *32*). TM6 opening in the β_2AR-Gi complex is very similar to the one observed in the β_2AR-Gs structure (Fig. 2E) and does not explain the increased efficacy of LM189 for Gαi recruitment. We used fluorescence spectroscopy to investigate the conformational dynamics induced by LM189 and other β_2AR ligands. For these studies, the receptor was labeled with monobromo-bimane at the cytoplasmic end of TM6 (mBBr-β_2AR) (*46*, *47*) (Fig. 5A). As previously observed, salmeterol-bound receptor induced an intermediate TM6 opening, while BI-167107 promoted a larger TM6 movement, which translated into a shift of the probe to a more polar and solvent-exposed environment, characterized by a drop in fluorescence intensity and a red shift in λ_{max} (*38*) (Fig. 5B). The LM189-bound receptor showed an even larger decrease in monobromo(trimethylammonio)bimane (mBBr) intensity, suggesting

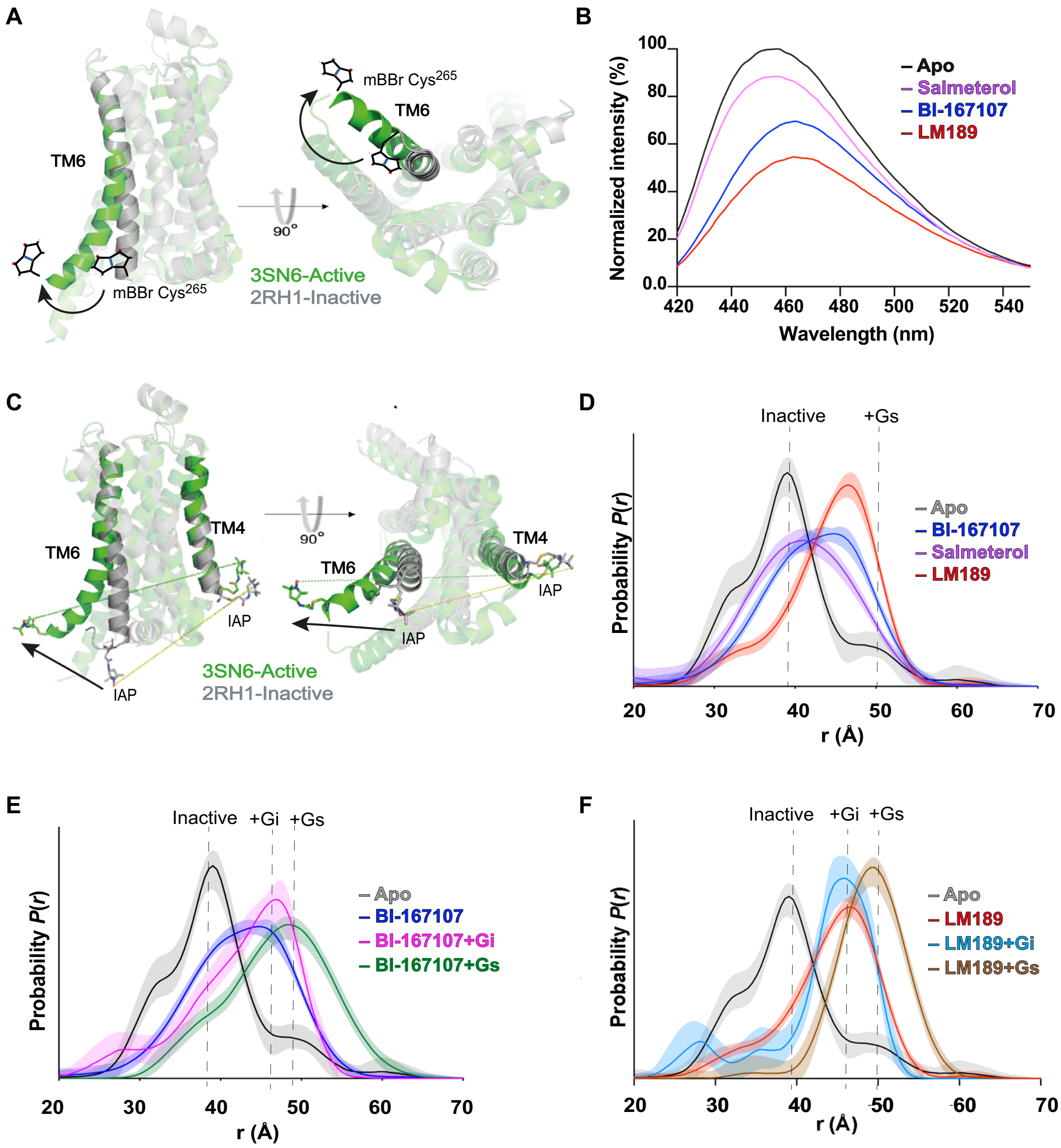

Fig. 5. Investigations of TM6 conformational dynamics. (**A** and **B**) Fluorescence spectroscopy measurements of β_2AR TM6 conformations. (A) Side and intracellular views of β_2AR labeled with mBBr on Cys265 of TM6. Inactive-state β_2AR (2RH1) (*44*) is colored gray, and active-state β_2AR (3SN6) (*4*) is colored green. (B) Steady-state fluorescence emission spectra of mBBr-labeled β_2AR purified in LMNG/CHS in the presence and absence of ligands. The spectra are normalized relative to apo (unliganded) receptor (gray). (**C** to **F**) TM4/6 DEER measurements of β_2AR purified in LMNG/CHS in the presence of ligands and G proteins. (C) Side and intracellular views of the receptor labeling sites on TM4 and TM6. Inactive-state β_2AR (2RH1) (*44*) is colored gray, and active-state β_2AR (3SN6) (*4*) is colored green. (D) Ligand dependence of TM4/6 distance distributions. (E) G protein dependence of distance distributions of BI-167107–bound receptor. (F) G protein dependence of distance distributions of the LM189-bound receptor. Distance distributions are color coded as indicated.

a larger TM6 opening compared to the full agonist BI-167107, or a larger fraction of the receptor in an active conformation (Fig. 5B). We observed similar results in experiments performed in lipid nanodiscs (HDL particles, fig. S5A), with a slightly less-pronounced shift in λ_{max} for LM189 compared to BI-167107. This suggests that the TM6 conformation stabilized by LM189 is different in its degree of opening and/or rotation from the conformations previously observed with other β_2AR agonists.

To better characterize LM189 conformational changes and their role in biased signaling and G protein specificity, we combined double electron-electron resonance (DEER) spectroscopy and single-molecule Förster resonance energy transfer (smFRET) investigations. For DEER studies, we used a minimal-cysteine version of β_2AR, spin labeled with iodoacetoamido-PROXYL (IAP) at the intracellular TM4/TM6 helices (N148C/L266C) (*17*) (Fig. 5C). The distance distribution for the unliganded (apo) receptor (gray in Fig. 5D and fig. S5, B to E) displays two main peaks centered at approximatively 32 and 39 Å as well as a smaller peak at 50 Å corresponding to a minor active-state population. Addition of the partial agonist salmeterol (purple in Fig. 5D and fig. S5, B and E) broadens the distribution and shifts the most probable distance to ~41 Å, indicative of a conformationally heterogeneous position of TM6 at an intermediate opening relative to both inactive and active distances. As previously reported (*17*), the ultrahigh-affinity full agonist BI-167107 (blue in Fig. 5D and fig. S5E) promoted a more open TM6 conformation (~45 Å) but failed to stabilize a fully outward active-state conformation observed in the β_2AR-Gs structure. Consistent with our bimane studies, we observed a greater TM6 outward movement in the presence of LM189, with a distance distribution dominated by a relatively narrow and monomodal peak at ~47 Å, in contrast with the broader distributions measured in the salmeterol and BI-167107 conditions (red in Fig. 5D and fig. S5E).

Next, we sought to evaluate the effect of the G proteins Gs and Gi on β_2AR distance distributions. Gi addition to salmeterol-coupled receptor resulted in a broad distance distribution, only marginally stabilizing the Gi-occupied conformation (fig. S5B). The incomplete shift to fully G protein–occupied receptor observed in the presence of salmeterol is likely due to the partial agonist efficacy of the ligand. In the presence of Gs, salmeterol-bound receptor exhibited a longer most probable distance, ~50 Å, in agreement with the BI-167107–β_2AR-Gs data (fig. S5, B and D). G protein coupling to BI-167107–bound receptor resulted in the stabilization of two distinct distance distributions for Gi and Gs, with most probable distances of ~47 and ~48 Å, respectively (Fig. 5E and fig. S5, C to E). These distributions are broader, more multimodal, and present greater probability density in the 30- to 40-Å range than those for the LM189-coupled receptor bound to G proteins. The addition of Gi to LM189-coupled β_2AR (~46 Å most probable distance) (light blue in Fig. 5F) populated a distance distribution with a great degree of overlap with that of the LM189-coupled receptor alone (red in Fig. 5, D and F, and fig. S5E). In contrast, Gs coupling to LM189-bound β_2AR resulted in a longer most probable distance (~49 Å) (brown in Fig. 5F), corresponding to a further ~3 Å shift compared to Gi-bound receptor. On the basis of these observations, LM189 may be Gi-biased because it stabilizes a TM6 outward conformation corresponding to the Gi-competent state, facilitating Gi recruitment. Conversely, Gs stabilizes a longer most probable distance, suggesting that TM6 populates slightly different conformations when the receptor is bound to Gi or Gs (Fig. 5, D to F, and fig. S5, B to E).

For smFRET studies, the minimal cysteine β_2AR construct was labeled at the intracellular TM4/TM6 with the donor and acceptor fluorophores DY549P1 and Alexa Fluor 647. Labeled β_2AR was subsequently surface immobilized and imaged using an objective–total internal reflection fluorescence (TIRF) microscope (*48*, *49*). Similar to our DEER results, the apo (unliganded) receptor mainly populated two high-FRET states centered at ~0.9 and 0.7 FRET, corresponding to the close proximity of helices TM4 and TM6, typical of the inactive receptor (black in Fig. 6, A and B). Individual FRET traces also showed rare excursions to lower FRET states, ranging from ~0.2 to ~0.6 FRET (black in Fig. 6B). Also, in agreement with our DEER data, upon addition of the full agonist BI-167107 (blue in Fig. 6, A and B), an intermediate state at ~0.6 FRET became the predominant population, at the expense of the inactive higher FRET states. We also observed a modest increase in the low-FRET values at ~0.2, suggesting the enhancement of the proportion of the receptor in the active state, where the donor and acceptor fluorophores on TM4 and TM6 are further apart (blue in Fig. 6, A and B). The smFRET states observed in the apo and BI-167107 conditions are in agreement with previous smFRET studies (*32*). However, compared to previous smFRET investigations on the β_2AR (*32*), we were able to resolve transitions between distinct receptor states (inactive, intermediate, and active) (Fig. 6, A and B). This was possible by using the donor/acceptor fluorophore pair DY549P1/Alexa Fluor 647, which combine a slightly longer Förster radius compared to the previously used Cy3B/Cy7 (*32*) with notably improved signal quality, mostly due to the increased brightness of Alexa Fluor 647 over Cy7.

Upon addition of the biased agonist LM189, we observed the coexistence of two major FRET populations: the intermediate one at ~0.6 FRET efficiency and a low-FRET state at ~0.3 FRET, which, based on our DEER measurements, we attribute to the outward TM6 conformation stabilized in the presence of LM189 (red in Figs. 5D and 6, A and B). The increase in the ~0.3 FRET state was at the expense of the intermediate-FRET population, which appeared to be less dominant for the LM189-bound receptor compared to the BI-167107 condition (Fig. 6A). While BI-167107 only has a minor tail in the low-FRET conformation, LM189 populates the ~0.3 FRET state ~40% of the time, even without the addition of G protein (Fig. 6A). This conformation corresponds to the Gi-competent state, as observed upon coupling of Gi in complex with scfv-16 to the LM189-bound receptor, shifting TM6 to the low-FRET peak (~0.3 FRET) almost completely (light blue in Fig. 6, B and C). Collectively, our DEER and smFRET data suggest that LM189 is a Gi-biased ligand because, unlike other agonists, it very effectively stabilizes the Gi-competent conformation, thereby facilitating Gi recruitment. While we observed one main peak in the DEER distance distribution for the receptor bound to LM189 (Fig. 5D), we detected two populations upon LM189 addition in our smFRET measurements (Fig. 6A). It is possible that the two conformations observed by smFRET exhibit subtle differences in TM6 position or local environment, and while these motions are amplified by the smFRET environmental sensitive dyes used in this study, they are not observable as TM4 to TM6 distance changes in DEER. This is also supported by our ensemble fluorescence experiments (Fig. 5B and fig. S5A), indicating that the LM189-stabilized TM6 conformation diverges in its degree of opening and helix rotation from the other ligands.

DISCUSSION

Despite the large number of structures available for GPCRs in complex with different G protein subtypes (*3*–*6*), the molecular determinants of G protein coupling specificity remain elusive. This is because

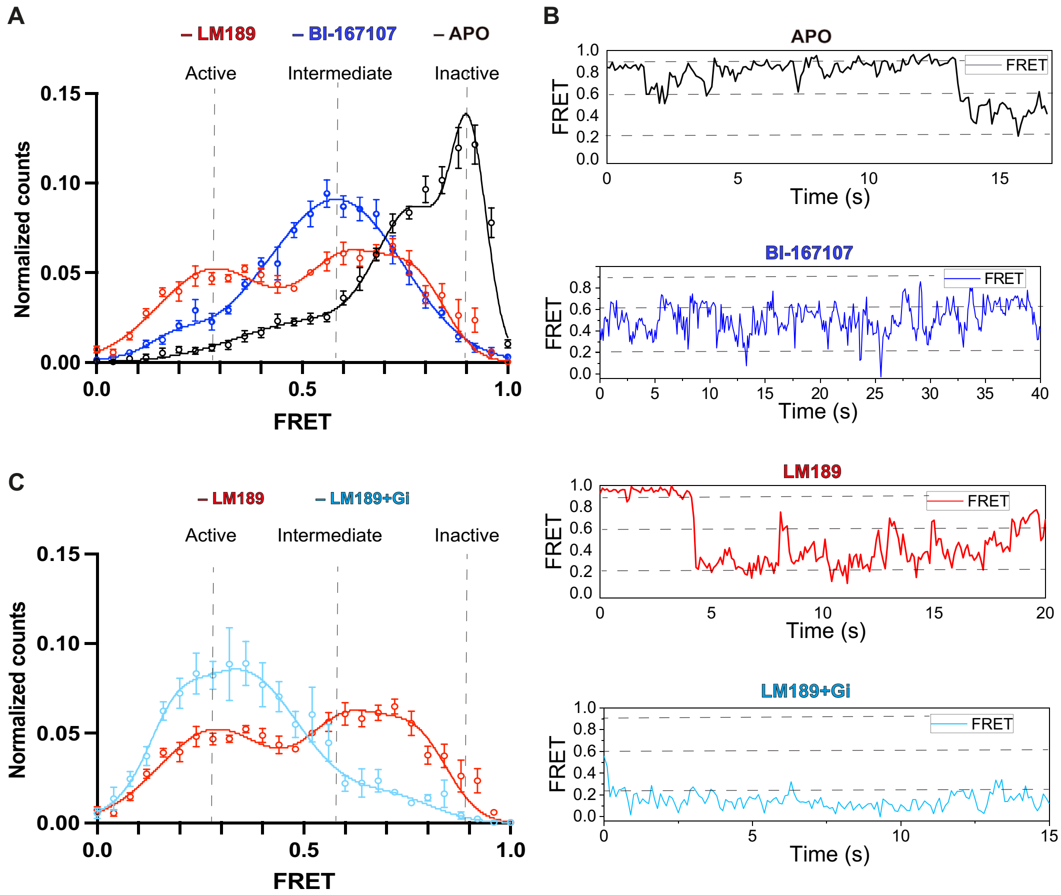

Fig. 6. SmFRET distributions of TM6 conformational dynamics. (**A** to **C**) SmFRET measurements of β_2AR labeled on TM4/6 with donor and acceptor fluorophores. (A) Unliganded (apo, $N = 269$) receptor, in black, mostly populates the inactive states (~0.9 and 0.7), corresponding to high FRET. BI-167107 (blue, $N = 265$), a β_2AR full agonist, mostly populates the intermediate state of the receptor (~0.6) and marginally stabilizes a state at ~0.2 FRET efficiency. The LM189-bound receptor (red, $N = 181$) populates the intermediate state at ~0.6 to a smaller degree, in favor of the low-FRET population (~0.3). (B) Representative FRET traces and transitions in the apo, BI-167107, LM189, and the LM189-bound receptor coupled to Gi and scfv-16. (C) Upon Gi coupling, the active state (~0.3), corresponding to low FRET, becomes predominant (light blue, $N = 105$).

specificity is hypothesized to be determined at the level of intermediate receptor conformations, which, due to their transient nature, are usually not accessible by traditional structural methods. In this context, GPCR agonists that are able to select specific signaling pathways, also called biased agonists, can drive the G protein coupling preference for a specific GPCR.

In this work, we aimed at a better understanding of the role of ligand efficacy and bias for G protein coupling specificity. We studied the binding of β_2AR to its main G protein, Gαs, and its secondary G protein, Gαi, using a combination of structural and biophysical methods. We identified salmeterol (*38*), a β_2AR partial agonist for Gs binding, as a full agonist for the recruitment of Gi with an efficacy greater than the native agonist epinephrine (Fig. 1, A to C). On the basis of the salmeterol scaffold, we developed LM189, a Gi-biased agonist according to our Glo assay and BRET investigations (Fig. 1 and fig. S1). For BRET assays, we used mini-G proteins, engineered G proteins that only contain the GTPase domains of Gα subunits. While the results obtained by BRET experiments are in agreement with our Glo assay investigations, it is important to highlight that the differences measured with the use of mini-G proteins do not necessarily reflect receptor selectivity in the context of more physiological systems, where fully reconstituted G protein heterotrimers better reflect receptor specificity profile.

LM189 was used to obtain the cryo-EM structure of the β_2AR-Gi complex (Fig. 2 and fig. S2). The orthosteric binding pocket of the LM189-β_2AR-Gi structure is very similar to the binding pocket of β_2AR in the presence of salmeterol and epinephrine (Fig. 3, A to C). This suggests that the G protein specificity mechanism exerted by the biased ligand LM189 is rather mediated by intermediate receptor conformations that involve the core and the intracellular cavity of the receptor. We observed the major differences between the β_2AR-Gi structure and the previously obtained β_2AR-Gs complex at the intracellular ICL2, TM6, and TM5 (Fig. 2E). Different conformations at ICL2, TM5, and TM6 have been reported in other GPCR–G protein complexes for receptors that can couple to both Gs and Gi, indicating that these intracellular domains might be involved in the initial recognition and engagement of distinct G protein subtypes (*7*, *8*, *33*). In the β_2AR-Gs complex, we observed a ~3-Å TM6 further opening compared to the Gi-bound structure (Fig. 2E). This structural difference has also been measured by DEER (Fig. 5, E and F). In agreement with previous GPCR–G protein structures, Gs and Gq,11,12,13 coupling is associated with a wider G protein-binding

pocket relative to Gi-coupled structures, to accommodate the bulkier C terminus of the α5 helix of the Gs and Gq isoforms and still allow the interaction with the less bulky Gi α5 helix (*9*, *13*, *50*). Because the size of the G protein binding pocket in the receptor intracellular core may reflect the receptor's ability to couple to multiple G proteins, it has been hypothesized that receptors that canonically couple to Gs (and Gq,11,12,13) are generally more promiscuous than those that are classified as Gi coupled (*11*). It should however be noted that the stable nucleotide-free β_2AR-Gs complex used in cryo-EM studies may contain structural changes in the G protein binding that are not observed in the transient nucleotide-free state in vivo (*51*). In addition, previous smFRET investigations have found evidence for at least one transient intermediate state in the process of complex formation (*32*). Therefore, biophysical studies done in the absence of G proteins provide more accurate information about the ligand-specific structural changes in the cytoplasmic surface that determine coupling specificity.

To investigate the receptor conformations at the basis of G protein specificity and the role of the biased ligand LM189, we conducted spectroscopic investigations at the intracellular ICL2 and TM6 of the receptor using CW-EPR, fluorescence spectroscopy, DEER, and smFRET. To note, these experiments were conducted in the absence of cmpd-6FA, the PAM used for structure stabilization of the β_2AR-Gi complex. CW-EPR investigations indicate that the loop-helix equilibrium of ICL2 is shifted toward the helical state to a greater degree by Gs binding compared with Gi binding (Fig. 4, B to D, and fig. S4, B to D), in agreement with previous NMR investigations (*14*). To note, when β_2AR was bound to LM189, we observed an increase in the mobile component of the CW-EPR spectra, a conformational transition that might be relevant for the initial stages of G protein recognition (Fig. 4B and fig. S4, B to D). In agreement with CW-EPR measurements, MD simulations at the LM189-β_2AR-Gi structure also show ICL2 unwinding upon removal of Gi, indicating that ICL2 conformational transitions may be involved in G protein coupling specificity (Fig. 3, F and G).

Fluorescence spectroscopy studies with a probe at the intracellular end of TM6 of β_2AR suggested the presence of a distinct conformation for LM189-occupied receptor compared to other nonbiased agonists, characterized by a larger TM6 outward movement (Fig. 5B and fig. S5A). DEER experiments showed that LM189-coupled β_2AR populates a notably similar TM4/TM6 distance distribution to that observed upon addition of the G protein Gi, suggesting that LM189 stabilizes the same receptor TM6 conformation as Gi (Fig. 5, D and F). This may explain the preferential Gαi recruitment observed in our experimental data by the biased ligand LM189 (Fig. 1 and fig. S1). In agreement with our structural work, which revealed a difference in rotation of the intracellular end of TM6 in the β_2AR-Gs structure compared to β_2AR-Gi (Fig. 2E), DEER measurements showed an additional ~3-Å TM6 structural change upon Gs binding (Fig. 5F), indicating slightly different conformations for receptor bound to different G protein subtypes (Fig. 5F and fig. S5, B to D). By smFRET, we detected a predominant intermediate state (~0.6 FRET) for the TM4 to TM6 labeling sites of β_2AR in the presence of the full agonist BI-167107 (Fig. 6, A and B). In contrast, we observed two FRET states for receptor bound to LM189, the intermediate-FRET state (~0.6 FRET) and a low-FRET state (~0.3 FRET), the latter populated even in the absence of the G protein (Fig. 6, A and B). Binding of the G protein Gi shifted the equilibrium of the LM189-bound receptor toward the ~0.3 FRET population (Fig. 6, B and C), corroborating our finding that LM189 is a Gi-biased agonist for its ability to stabilize the Gi-competent conformation.

In this study, we observed that, relative to nonbiased agonists, the biased ligand LM189 stabilizes a distinct, specific TM6 conformation (Figs. 5, B and D, and 6A) and increases ICL2 dynamics (Fig. 4, B to D). Altogether, our investigations reveal that distinct conformations at ICL2 and TM6 of β_2AR are required for the binding of the different G protein subtypes Gαs and Gαi, underlying the importance of receptor conformational dynamics for coupling specificity and promiscuity. Altogether, these results deepen our understanding of G protein specificity and bias and can be useful in the design of ligands that select for preferred signaling pathways.

MATERIALS AND METHODS

Expression and purification of the β_2AR

The β_2AR construct named PN1 was used for all experiments except EPR, DEER, and smFRET investigations. PN1 was expressed and purified as previously described (*35*). Briefly, receptor was expressed in *Spodoptera frugiperda* (*Sf9*) insect cells (Expression Systems, cell line IPLB-Sf-21-AE, catalog number 94-001S) using the baculovirus method, and media was supplemented with 1 μM alprenolol. Cells expressing β_2AR were harvested and lysed as previously described (*35*). The receptor was solubilized from membranes using 20 mM hydroxy-ethylpiperazine ethane sulfonic acid (Hepes), pH 7.4, 100 mM sodium chloride (NaCl), 1% *n*-dodecyl-β-D-maltopyranoside (DDM), 0.03% cholesteryl hemisuccinate (CHS), 2 mM $MgCl_2$, 1 μM alprenolol, and protease inhibitors. Membranes were homogenized with a douncer and the soluble fraction was isolated by centrifugation and applied to an M1 anti-FLAG immunoaffinity resin. The receptor bound to the resin was extensively washed with 20 mM Hepes, pH 7.4, 350 mM NaCl, 0.1% DDM, 0.01% CHS, 10 μM alprenolol, and protease inhibitors to lower the detergent concentration. To exchange detergent from 0.1% DDM/0.01% CHS to 0.01% (w/v) lauryl maltose neopentyl glycol (LMNG, Anatrace)/0.001% CHS, the receptor was extensively washed with a progressive gradient of DDM: LMNG buffer. In parallel, while the receptor was bound to the resin, alprenolol was removed by washing with saturating concentrations of the low-affinity antagonist atenolol. Because of the fast dissociation kinetics of atenolol from the β_2AR, subsequent washes with ligand-free buffer yielded unliganded β_2AR. The receptor was then eluted in a buffer consisting of 20 mM Hepes, pH 7.4, 150 mM NaCl, 0.01% LMNG/0.001% CHS, FLAG peptide, and 5 mM EDTA. Receptor was further purified by size exclusion chromatography (SEC; Superdex 200 10/300 gel filtration column) in buffer containing 20 mM Hepes, pH 7.4, 150 mM NaCl, and 0.01% LMNG/0.001% CHS. Last, β_2AR was concentrated to 250 μM, flash frozen after the addition of 20% glycerol, and stored in −80°C.

As we did not perform an additional purification step using alprenolol resin, the functional fraction of purified receptor was assessed by direct coupling of purified β_2AR to the cognate G protein Gs. Immediately after purification and SEC, PN1 was labeled with mBBr in the presence of 100 μM tris(2-carboxyethyl)phosphine (TCEP). Excess dye was removed by SEC and the labeled receptor was incubated with 10× molar ratio of the agonist ligand BI-167107 for 30 min. Subsequently, a 1:1 ratio of Gs protein was added for 1 hour, followed by overnight treatment with apyrase (1 unit, NEB) on ice. Following SEC, 80% of the receptor purified in 0.1% DDM, 0.01% CHS, and 90% of β_2AR in 0.01% LMNG/0.001% CHS was

attested to be functional, as capable to bind to Gs and to run as a single mono-disperse peak on SEC.

To favor Gi coupling, we avoided working with buffers containing CHS (*35*). To perform the experiments in this work, β_2AR was extensively diluted into buffers not containing CHS or reconstituted onto HDLs along with neutrally charged lipids. For cryo-EM purposes, β_2AR was purified in 20 mM Hepes, pH 7.4, 150 mM NaCl, 0.1% DDM, and 0.01% CHS as previously described and subsequently exchanged to LMNG/cholesterol micelles to avoid the use of CHS. LMNG [5% (w/v)] and 2 mol % cholesterol were prepared by overnight stirring followed by sonication in buffer containing 200 mM Hepes, pH 7.4. While bound to M1-Flag during purification, β_2AR was buffer exchanged onto 20 mM Hepes, pH 7.4, 150 mM NaCl, and 0.01% LMNG/0.003% cholesterol micelles. After the exchange, the receptor was washed with atenolol and eluted from the M1-Flag. All subsequent steps took place as previously described, using LMNG/cholesterol buffers instead of LMNG/CHS.

Expression, purification, and labeling of the β_2AR for CW-EPR and DEER experiments

For CW-EPR experiments, we used the β_2AR minimal cysteine background construct called β2Δ5, which has the following mutations: C77V, C265A, C327S, C378A, and C406A (*17*). In addition, β2Δ5 has an N-terminal FLAG sequence and a hexahistidine sequence at the C terminus, as well as methionine substitutions M96T and M98T to increase expression levels.

For DEER investigations, we used the β_2AR minimal cysteine background construct β2Δ6, which carries an extra mutation at C341L, removing the palmitoylation site. Cysteine mutations at desired locations were reintroduced in the β2Δ5 and β2Δ6 backgrounds to site-specifically label these constructs for CW-EPR and DEER. To monitor ICL2 rearrangements by CW-EPR, Q142C on ICL2 was inserted into a β2Δ5 background. For DEER investigations, we used the previously established β2Δ6 148C/266C construct, with reintroduced cysteines at residues N148C and L266C (*32*). The single-cysteine mutants N148C and L266C were also produced for control experiments to monitor nitroxide probe mobility and optimal labeling conditions.

All β2Δ5 and β2Δ6 constructs used for EPR and DEER were cloned into the pcDNA-Zeo-tetO vector as previously described (*22*). Constructs were transfected into the suspension cell line tetracycline-inducible Expi293 cells (Thermo Fisher Scientific, catalog number A14635), stably expressing the tetracycline repressor (*52*). Expifectamine transfection kit (Thermo Fisher Scientific) was used to transfect the cells according to the manufacturer's recommendations. Two days posttransfection, receptor expression was induced with doxycycline (4 mg/ml, 5 mM sodium butyrate) in the presence of 2 μM alprenolol. Thirty hours postinduction, the cells were harvested by centrifugation and the pellet was frozen in liquid nitrogen and stored at −80°C. The receptor was subsequently purified in DDM/CHS and exchanged to LMNG/CHS as described above. After elution from the M1-Flag column, the receptor was labeled with the spin label reagent IAP in the presence of 100 μM TCEP in buffer containing 20 mM Hepes, pH 7.4, 150 mM NaCl, and 0.01% LMNG/0.001% CHS. Twenty-five–fold molar excess of IAP was added to 40 μM β2Δ6 receptor for 3 hours at room temperature (RT). For β2Δ5, 10-fold molar excess of IAP was added for 2 hours at RT. After quenching of the reaction with 5 mM final L-cysteine, the receptor was separated from the excess spin label by SEC (Superdex 200 10/300 gel filtration column) in SEC buffer (20 mM Hepes, pH 7.4, 150 mM NaCl, and 0.01% LMNG) prepared with D_2O and not containing CHS.

Expression, purification, and labeling of the β_2AR for smFRET experiments

The β2Δ6 148C/266C construct was used for smFRET experiments. Expression and purification of β2Δ6 148C/266C were carried out as described for the DEER experiments. For smFRET labeling, receptor eluted from M1-Flag was diluted to 10 μM in 20 mM Hepes, pH 7.4, 150 mM NaCl, and 0.01% LMNG/0.001% CHS, and 100 μM TCEP was added for 20 min. Labeling was conducted in the presence of 2 μM atenolol and was initiated by the addition of 10× of the premixed fluorescent dye pair DY559P1 and Alexa Fluor 647, at 1:1 ratio. After 1 hour at RT, the reaction was quenched with 5 mM L-cysteine. Further sample purification and TCEP and dye removal were performed by SEC (Superdex 200 10/300 gel filtration column) in 20 mM Hepes, pH 7.4, 150 mM NaCl, and 0.01% LMNG buffer. Pulled fractions were concentrated to 15 μM and frozen in liquid nitrogen with 20% glycerol for subsequent smFRET experiments.

Expression, purification, and labeling of the β_2AR receptor for fluorescence spectroscopy

We used the PN1 construct for steady-state ensemble fluorescence experiments. PN1 was expressed in *sf9* cells, purified and exchanged to LMNG as previously described (*35*). For labeling, 100 μM TCEP was added for 20 min to 10 μM PN1 in 20 mM Hepes, pH 7.4, 150 mM NaCl, and 0.01% MNG buffer. Labeling was initiated by the addition of 20× mBBr for 45 min at RT. The reaction was quenched with excess of L-cysteine and the sample was further purified by SEC (Superdex 200 10/300 gel filtration column) in 20 mM Hepes, pH 7.4, 150 mM NaCl, and 0.01% LMNG buffer. Purified and labeled β_2AR fractions were concentrated and flash-frozen for subsequent experiments or for reconstitution into HDLs.

HDL reconstitution

β_2AR was reconstituted into HDL particles as previously described (*35*). Briefly, receptor was mixed with the MSP1D1 belt protein in a 1:10 receptor:MSP molar ratio and with lipids (3:2 POPC/POPE or POPC/POPG) in a 1:40 MSP:lipids molar ratio. After 2 hours of incubation at 4°C, Biobeads (Bio-Rad) were added at a ratio of 1:10 lipids:beads and incubated for 4 hours at 4°C to remove the detergent. Upon Biobeads removal by centrifugation, empty discs were separated from β_2AR-containing discs by M1-flag affinity chromatography and subsequent SEC (Superdex 200 10/300 gel filtration column) in 20 mM Hepes, pH 7.4, and 100 mM NaCl. HDLs were concentrated to 30 μM, flash-frozen in liquid nitrogen, and stored at −80°C for future use.

Expression and purification of heteromeric $G\alpha_1\beta_1\gamma_2$

Heterotrimeric Gi was expressed and purified as previously described (*3*) with some modifications. Briefly, heterotrimeric Gi was expressed in *Trichoplusia ni* insect cells using the baculovirus method (Expression Systems, catalog number 94-002S). Two viruses were used to infect the insect cells, one encoding the wild-type human $G\alpha_1$ subunit and another one encoding the wild-type human $\beta_1\gamma_2$ subunits. The cells were harvested 48 hours posttransfection and the pellet was flash-frozen with liquid nitrogen and stored at −80°C. Cells lysis was conducted in 10 mM tris, pH 7.4, 100 mM

magnesium chloride ($MgCl_2$), 5 mM β-mercaptoethanol (βME), 20 mM GDP, and protease inhibitors. Membranes were isolated by centrifugation and solubilized using a douncer in 20 mM Hepes, pH 7.4, 100 mM NaCl, 1% sodium cholate, 0.05% DDM, 5 mM magnesium chloride, 5 mM βME, 20 mM GDP, 20 U calf intestinal alkaline phosphatase, and protease inhibitors. After the addition of 20 mM imidazole, the solubilization mixture was stirred for 1 hour at 4°C. After centrifugation, the supernatant was loaded on a Ni-NTA chromatography column, extensively washed in 0.05% DDM buffer to remove the cholate. Subsequently, a progressive gradient of DDM:MNG buffer was used to exchange the detergent from DDM to 0.05% LMNG. After elution in the presence of 250 mM imidazole, the purified heterotrimer was dephosphorylated by lambda protein phosphatase (NEB), calf intestinal phosphatase (NEB), and antarctic phosphatase (NEB) in the presence of 1 mM $MnCl_2$ for 1 hour on ice. Gi heterotrimer was separated from excess betagamma using a MonoQ 10/100 GL column (GE Healthcare). The protein was diluted to lower the imidazole concentration and loaded onto the column in 20 mM Hepes, pH 7.4, 50 mM NaCl, 1 mM $MgCl_2$, 0.05% LMNG, 100 mM TCEP, and 20 mM GDP. The heterotrimer was eluted with a linear gradient of 0% to 50% with buffer containing 1 M NaCl. Eluted fractions were concentrated to 200 to 250 μM, and after the addition of 20% glycerol, the protein was flash-frozen and stored at −80°C.

Development and synthesis of LM189

NMR analyses

The analytical characterization of LM-189 was performed by ^{1}H-NMR at 600 MHz and ^{13}C-NMR at 151 MHz. Determination of chemical shifts [parts per million (ppm)] was done in relation to the solvent used (CD_3OD). The NMR samples were measured under nitrogen atmosphere to avoid decomposition of the catecholaminergic product. The usual abbreviations are used for signal multiplicities: s (singlet), d (doublet), t (triplet), q (quartet), quint (quintet), sext (sextet), and sept (septet). Coupling constants are given in hertz.

Polarimetry

Specific optical rotations were measured using a JASCO P-2000 polarimeter. Chamber path length: 100 mm, chamber volume: maximum 1.2 ml. The target compound was measured in the form of a clear solution in methanol.

UHPLC-MS

Liquid chromatography–mass spectrometry (LC-MS) analyses were conducted using a Dionex UltiMate 3000 UHPLC system with an RS diode array detector for the wavelengths 220 and 254 nm. A binary solvent system (mixture of 0.1% aqueous formic acid and methanol) was used as the eluent. Column: Kinetex C8 (75 × 2.1 mm, 2.6 μm, 0.3 ml/min flow rate) or ZORBAX ECLIPSE XDB-C8 (100 × 3.0 mm, 3.5 μm, 0.4 ml/min flow rate). Mass detection was performed on a Bruker Amazon SL mass spectrometer [electron spray ionization (ESI)].

High-resolution MS

High-resolution MS (HRMS) measurements were performed on a Bruker timsTOF Pro device.

Preparative RP-HPLC

The target compound was purified by preparative, reverse-phase HPLC, applying mixtures of 0.1% aqueous trifluoroacetic acid and acetonitrile as organic component. The separations were conducted on a preparative HPLC AGILENT SERIES 1100 system equipped with a variable wavelength detector (VWD), on an AGILENT HPLC 1260 Infinity system with a VWD or on an AGILENT HPLC 1260 Infinity II system with a VWD. Column: ZORBAX ECLIPSE XDB-C8 (150 × 21.5 mm, 5 μm).

Analytical RP-HPLC

Analytical HPLC runs for purity control were conducted on an AGILENT 1200 series with DAD detector and peak detection at 220, 230, 254, and 280 nm. The used column was a ZORBAX ECLIPSE XDB-C8 (100 × 4.6 mm, 5 μm) with a flow rate of 0.5 ml/min. The column thermostat was set to 20°C to obtain uniform results. The binary solvent systems either consisted of 0.1% aqueous TFA + acetonitrile or 0.1% aqueous TFA + methanol. The following two eluent systems were used:

System 1A: 0.1% aqueous trifluoroacetic acid + acetonitrile, 0.5 ml/min: 5% acetonitrile from 0 to 3 min, to 95% at 18 min, 95% from 18 to 24 min, to 5% at 27 min, 5% from 27 to 30 min.

System 1B: 0.1% aqueous trifluoroacetic acid + methanol, 0.5 ml/min: 5% methanol from 0 to 3 min, to 95% at 18 min, 95% from 18 to 24 min, to 5% at 27 min, 5% from 27 to 30 min.

(*R*)-4-(1-hydroxy-2-((6-(4-phenylbutoxy)hexyl)amino)ethyl)benzene-1,2-diol × TFA (LM-189)

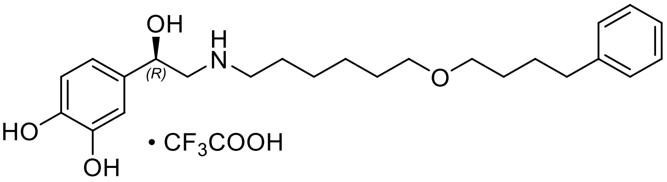

(4-((6-Bromohexyl)oxy)butyl)benzene (66.0 mg, 0.21 mmol) was dissolved in dimethyl sulfoxide (DMSO; 1 ml) in a microwave tube. To this solution, (*R*)-norepinephrine freebase (100 mg, 0.59 mmol) was added. The vial was set under nitrogen atmosphere, sealed with an aluminum crimp cap and heated to 70°C for 7 hours under light protection. Then, the reaction was stirred at RT overnight (18 hours) and directly added to a stoichiometric excess of 0.3% TFA solution. The mixture was frozen and lyophilized. The crude compound was purified by preparative HPLC (ZORBAX ECLIPSE XDB-C8, 0.1% TFA + 10% acetonitrile to 95% acetonitrile at 10 min, peak eluted at 7 min) to give the target compound LM-189 as a yellow-orange oil (59.0 mg, 55% yield).

Analytical data were in agreement with the literature (racemate of LM-189) (*53*, *54*).

$[\alpha]_D^{26}$ −17.8 (c = 1.27 in methanol)

ESI-HRMS Calculated for $C_{24}H_{36}NO_4$: mass/charge ratio (m/z) 402.2639 $[M + H]^+$

Found: m/z 402.2638 $[M + H]^+$.

^{1}H NMR (600 MHz, CD_3OD) δ (ppm): 7.25–7.21 (m, 2H, *meta*-H phenyl), 7.16 (d, J = 7.4 Hz, 2H, *ortho*-H phenyl), 7.13 (t, J = 7.3 Hz, 1H, *para*-H phenyl), 6.86 (d, J = 1.5 Hz 1H, catechol H3), 6.77 (d, J = 8.1 Hz, 1H, catechol H6), 6.73 (dd, J = 8.1, 1.5 Hz, 1H, catechol H5), 4.79 (dd, J = 9.9, 3.2 Hz, 1H, C<u>H</u>OH), 3.44 (t, J = 6.5 Hz, 2H, phenylbutyl hexyl ether C<u>H2</u>O), 3.42 (t, J = 6.5 Hz, 2H, phenylbutyl hexyl ether C<u>H2</u>O), 3.11 (dd, J = 12.5, 3.4 Hz, 1H, CH(OH)C<u>H</u>$_2$N), 3.06 (dd, J = 12.5, 10.0 Hz, 1H, CH(OH)C<u>H</u>$_2$N), 3.01 (t, J = 8.0 Hz, 2H, hexylene NHC<u>H2</u>) 2.62 (t, J = 7.6 Hz, 2H, phenylbutyl PhC<u>H2</u>), 1.74–1.64 (m, 4H, phenylbutyl hexyl ether C<u>H2</u>),

1.61–1.55 (m, 4H, phenylbutyl hexyl ether C<u>H2</u>), 1.44–1.39 (m, 4H, phenylbutyl hexyl ether C<u>H2</u>).

^{13}C NMR (151 MHz, CD_3OD) δ (ppm): 146.7, 146.6, 143.7, 133.7, 129.4, 129.3, 126.7, 118.5, 116.4, 114.1, 71.8, 71.6, 70.0, 55.3, 48.9, 36.7, 30.5, 30.3, 29.3, 27.4, 27.0, 26.8.

HPLC t_R = 16.4 min (system 1A), purity 97% (254 nm), 98% (280 nm).

t_R = 11.7 min (system 1B), purity 98% (254 nm), 98% (280 nm).

GTP turnover assay

The GTP turnover assay was adapted from the GTPase-Glo assay (Promega) as described previously (*32*). To monitor Gs turnover, PN1 in 0.01% LMNG (75 nM final) was incubated for 1 hour at RT with 10× ligand excess in in 20 mM Hepes, pH 7.5, 100 mM NaCl, 0.01% MNG, and 20 μM GTP. When complexing with Gi, PN1 was used at 1 μM final concentration. For HDL experiments, we used 300 nM receptor in discs in 20 mM Hepes, pH 7.5, 100 mM NaCl, and 20 μM GTP. To start the reaction, G protein (1 μM for Gi AND 0.5 μM for Gs) in buffer containing 20 mM Hepes, pH 7.4, 100 mM NaCl, 20 mM $MgCl_2$, 200 μM TCEP, 0.01% LMNG, and 20 μM GDP was added to receptor in MNG. For HDL experiments, we used 0.5 μM G protein in 20 mM Hepes, pH 7.4, 100 mM NaCl, 20 mM $MgCl_2$, 200 μM TCEP, 0.04% DDM, AND 20 μM GDP. The reaction was carried over for 1 hour to monitor Gi turnover and for 10 min for Gs. After incubation at RT, GTPase-Glo reagent supplemented with 10 mM adenosine 5′-diphosphate (ADP) was added to the reaction and incubated for 30 min at RT. Detection reagent was then added and incubated for 10 min at RT before luminescence detection using a MicroBeta^2 microplate counter (PerkinElmer). Experiments were performed as biological triplicates and results were plotted using GraphPad Prism. P values were calculated using the unpaired t test analysis on GraphPad Prism, assuming Gaussian distributions. NS = $P > 0.05$, *$P \leq 0.05$, **$P \leq 0.01$, ***$P \leq 0.001$, and ****$P \leq 0.0001$.

Radioligand binding assay with membranes from HEK cells

Binding affinities toward the human β_1AR and β_2AR were determined as described previously (*55*, *56*). Human embryonic kidney (HEK) 293T cells were transiently transfected with the cDNA for β_1AR and β_2AR (obtained from the cDNA Resource Center, www.cdna.org). Membranes were prepared showing receptor densities of 3.2 pmol/mg protein (B_{max} for β_1AR) and 2.3 ± 0.64 pmol/mg protein (B_{max} for β_2AR) and binding affinities for the radioligand [^{3}H]CGP12,177 (specific activity, 51 Ci/mmol; PerkinElmer, Rodgau, Germany) of 0.070 nM (K_d for β_1AR) and 0.095 ± 0.02 nM (K_d for β_2AR). Competition binding experiments were performed by incubating membranes in binding buffer (25 mM Hepes, 5 mM $MgCl_2$, 1 mM EDTA, and 0.006% bovine serum albumin at pH 7.4) at a final protein concentration of 3 to 10 μg per well, together with the radioligand (final concentration, 0.2 nM) and varying concentrations of the competing ligands for 60 min at 37°C. Nonspecific binding was determined in the presence of unlabeled CGP12,177 at a final concentration of 10 μM. Protein concentration was established using the method of Lowry *et al.* (*57*). For data analysis the resulting competition curves were analyzed by nonlinear regression using the algorithms implemented in PRISM 10.0 (GraphPad Software, San Diego, CA) to provide an IC_{50} value, which was subsequently transformed into a K_i value using the equation of Cheng and Prusoff (*58*) (table S1). Mean K_i values were calculated from 7 to 11 single experiments each performed in triplicates.

β-Arrestin-2 recruitment (PathHunter assay)

Determination of receptor-stimulated β-arrestin-2 recruitment was performed applying the PathHunter assay (DiscoverX, Birmingham, UK), which is based on the measurement of fragment complementation of β-galactosidase as previously described (*55*, *59*). In detail, HEK293T cells stably expressing the enzyme acceptor tagged β-arrestin-2 fusion protein were transfected with the cDNA for β_1AR and β_2AR, each fused to the ProLink-PK1 fragment for enzyme complementation and transferred into 384-well microplates. Measurement started by incubating cells with epinephrine or LM189 for 90 min and was stopped by the addition of the detection mixture. Chemoluminescence was monitored with a Clariostar plate reader (BMG, Ortenberg, Germany) and analyzed by normalizing the raw data relative to basal activity (0%) and the maximum effect of norepinephrine (100%). Dose-response curves were analyzed applying the algorithms for four-parameter nonlinear regression implemented in Prism 10.0 (GraphPad LLC, CA) to yield EC_{50} and E_{max} values (table S1). Mean EC_{50} and E_{max} values were calculated from 6 to 17 independent experiments each conducted in duplicate.

Formation and purification of the β_2AR-Gi-scfv16 complex for cryo-EM

PN1 in 0.01% LMNG/2 mol % cholesterol was incubated with sevenfold molar excess of LM189 ligand for 30 min at RT. Cmpd-6FA PAM (*42*, *43*) was then added at sevenfold molar excess and incubated for an additional 30 min at RT. A 1.3-fold molar excess of Gi was added together with 100 μM TCEP and incubated for 2 hours at RT. Afterward, 2-fold molar excess of scfv16 was added to the complex and incubated for 1.5 hours on ice. Apyrase (1-unit, NEB) was then added, and the complex was incubated overnight on ice. The following day, the complex was diluted in 20 mM Hepes, pH 7.4, 100 mM NaCl, 0.01% MNG/2 mol % cholesterol, 0.0033% GDN, 10 μM LM189, 1 μM cmpd-6FA, and 3 mM Ca^{2+} and loaded onto M1 anti-FLAG affinity chromatography. Detergent concentration was lowered by washing with buffer containing 20 mM Hepes, pH 7.4, 100 mM NaCl, 0.001% MNG/2 mol % cholesterol 0.00025% GDN, 2 mM Ca^{2+}, 10 μM ligand, and 1 μM cmpd-6FA. Complex was eluted in 20 mM Hepes, pH 7.4, 100 mM NaCl, 0.00075% MNG/2 mol % cholesterol, 0.00025% GDN, FLAG peptide, 5 mM EDTA, 10 μM LM189, and 1 μM cmpd-6FA. TCEP (100 μM), 3 mM $MgCl_2$, and 1:1 (Gi:scfv) molar ratio of scfv16 were added to the complex immediately after elution. Free receptor was separated from the complex by SEC on a Superdex 200 10/300 increase column in 20 mM Hepes, pH 7.4, 100 mM NaCl, 0.00075% MNG/2 mol %, 0.00025% GDN, FLAG peptide, 5 mM EDTA, 1 μM LM189, and 0.1 μM cmpd-6FA. Peak fractions were concentrated to 15 to 20 mg/ml, filtered, and used for EM experiments.

Cryo-EM data collection and processing

Three-microliter aliquot of the β2AR-Gi-scfv16 complex was applied onto glow-discharged 300 mesh grids (Ultraufoil R1.2/1.3 or Quantifoil R1.2/1.3) and vitrified using a Vitrobot Mark IV (Thermo Fisher Scientific) under 100% humidity and 4°C conditions. Cryo-EM data were collected on a Titan Krios electron microscope operating at 300 kV and equipped with a K3 direct electron detector. Movies

were acquired with a calibrated pixel size of 1.111 Å/pixel and a total dose of ~52.5 electrons/Å^2, fractionated across 50 frames (fig. S2 and table S2).

Data processing was performed using RELION 3.1.2 and cryoSPARC 3.3.2 (*60*). Initially, motion correction was carried out on the movies using RELION's built-in implementation, followed by contrast transfer function (CTF) estimation using CTFFIND4 (*61*). Reference-based particle picking used previously determined GPCR–G protein 2D classes. For the ultrafoil grid dataset, 3,659,953 particles were picked, subjected to 2D classification to remove low-quality particles, and further sorted through two rounds of 3D classification. This process yielded 265,559 particles and a 3.4-Å-resolution structure after 3D refinement. In the case of the quantifoil grid dataset, 5,327,360 particles were picked, followed by one round of 2D classification and 3D classification, resulting in 513,747 particles and a 3.4-Å-resolution structure. The two datasets were then merged, and a 3D classification without image alignment was performed, leaving 477,875 particles and a 3.3-Å-resolution structure after 3D refinement.

Subsequent steps included CtfRefine, particle polishing, and the application of a mask to exclude the micelle and flexible α-helical domain. The final structure reached 2.9 Å resolution. The particles were then imported into cryoSPARC for nonuniform refinement, and the resulting map was sharpened using the Phenix autosharpen function to enhance map quality (*62*). Last, local resolution estimation and 3D Fourier shell correlation (3DFSC) were used to assess the local resolution and orientation distribution of the final dataset (*63*) (fig. S2).

Model building and refinement

The individual structures of β_2AR, Gi heterotrimer, and scfv16 were independently docked into the final sharpened map. Model and geometric restraints for LM189 and cmpd-6FA were generated using the Phenix elbow tool (*64*). In addition, four cholesterol molecules were built into densities corresponding to previously identified cholesterol binding sites (*65*). The model was iteratively refined and validated through multiple rounds of Phenix real-space refinement and manual refinement in Coot (table S2) (*66*).

MD simulations

Simulations of the β_2AR-Gi complex were based on the herein reported LM189-bound β_2AR-Gi cryo-EM. The ligand LM189 was either kept in the orthosteric binding site or replaced by epinephrine by structurally aligning the cryo-EM with the epinephrine-bound β_2AR-Nb6B9 structure [Protein Data Bank (PDB) ID: 4LDO] (*67*) and transferring the coordinates of epinephrine. Simulations of the β_2AR-Gs complex were based on the BI-167107–bound β_2AR-Gs crystal structure (PDB ID: 3SN6) (*4*). To obtain the LM189-bound and epinephrine-bound β_2AR-Gs complexes, the coordinates of BI-167107 were removed and replaced by the coordinates of LM189 and epinephrine after structurally aligning the herein reported LM189-bound β_2AR-Gi cryo-EM or the epinephrine-bound β_2AR-Nb6B9 structure (PDB ID: 4LDO) onto the β_2AR crystal structure, respectively. For further comparison, the salmeterol-bound β_2AR-Nb71 structure (PDB ID: 6MXT) was subjected to MD simulations.

The five receptor complexes (LM189-β_2AR-Gi, LM189-β_2AR-Gs, epinephrine-β_2AR-Gs, epinephrine-β_2AR-Gi, and salmeterol-β_2AR-Nb71) were further prepared using UCSF Chimera (*68*). To save computational resources, we conducted all MD simulations without intracellular proteins but applied position restraints on all receptor residues within 5 Å of the G protein or the Nb71 to maintain the respective conformation of the β_2AR. The three missing amino acids 176 to 178 in the ECL2 of the β_2AR-Gs crystal structure (PDB ID: 3SN6) were modeled by means of MODELLER (*69*), hydrogens were added, auxiliary proteins were removed, and chain termini were capped with neutral acetyl and methylamide groups. Except for Asp$^{2.50}$ and Glu$^{3.41}$, all titratable residues were left in their dominant protonation state at pH 7.0. Asp$^{2.50}$ has been suggested to be protonated in the active state (*70*), and residue Glu$^{3.41}$ directly contacts the lipid interface and therefore will also exist predominantly in its protonated state (*71*, *72*). Thus, these residues were protonated in MD simulations. LM189, epinephrine, and salmeterol were protonated at the secondary amine allowing the formation of the canonical salt bridge to Asp$^{3.32}$ conserved in aminergic GPCRs.

Parameter topology and coordinate files of the four receptor complexes were built up using the leap module of AMBER18 (*73*). Parameters for ligands were assigned using antechamber (*73*). Therefore, the structures of LM189, epinephrine, and salmeterol were optimized by means of Gaussian16 (*74*) at the B3LYP/6-31G(d) level (attributing a formal charge of +1), charges were calculated at the HF/6-31G(d) level, and atom point charges were assigned according to the restrained electrostatic potential procedure (*75*). Energy minimization was performed applying 500 steps of steepest decent followed by 4500 steps of conjugate gradient. The protein structures were aligned to the Orientation of Proteins in Membranes (*76*) Gs-bound structure of β_2AR (PDB ID: 3SN6). Each complex was inserted into a pre-equilibrated membrane of dioleyl-phosphatidylcholine (DOPC) lipids by means of the GROMACS tool g_membed (*77*). Subsequently, water molecules were replaced by sodium and chloride ions to give a neutral system with 0.15 M NaCl. The final system dimensions were roughly 80 Å by 80 Å by 100 Å (*68*), containing about 156 lipids, 58 sodium ions, 67 chloride ions, and about 13,200 water molecules. For all simulations, the general AMBER force field (*78*) (GAFF2) was used for ligands, the lipid14 force field (*79*) for DOPC molecules, and ff14SB (*80*) for the protein residues. The SPC/E water model (*81*) was applied.

Simulations were performed using GROMACS 2021.1 (*82*–*84*). The simulation systems were energy minimized and equilibrated in the NVT ensemble at 310 K for 1 ns followed by the NPT ensemble for 1 ns with harmonic restraints of 10.0 kcal·mol^{-1} on protein and ligands. In the NVT ensemble, the V-rescale thermostat was used. In the NPT ensemble, the Berendsen barostat, a surface tension of 22 dyn·cm^{-1} and a compressibility of 4.5×10^{-5} bar^{-1} were applied. During the equilibration and the subsequent productive MD runs, position restraints of 10.0 kcal·mol^{-1} were applied on the β_2AR residues within 5 Å of the G protein interface.

Multiple simulations were started from the final snapshot of the equilibration procedure for each of the four receptor complexes, resulting in productive MD simulation runs of 3 × 2 μs for each simulation system. Simulations were performed using periodic boundary conditions and a time step of 2 fs with bonds involving hydrogen constrained using LINCS (*85*). Long-range electrostatic interactions were computed using particle mesh Ewald method (*86*) with interpolation of order 4 and a fast Fourier transform (FFT) grid spacing of 1.6 Å. Nonbonded interactions were cut off at 12.0 Å. The analysis of the trajectories was performed using the CPPTRAJ module (*87*) of AMBER18. Interaction frequencies, distances, and dihedrals were plotted using Matplotlib 2.2.2 (*88*).

The equilibrated LM189-bound β_2AR-Gi and epinephrine-bound β_2AR-Gs complexes were further subjected to unrestrained MD simulations. In these simulations, following the same protocols outlined above,

the position restraints on residues near the G protein interface were removed to allow for greater conformational flexibility. For the epinephrine-bound β$_2$AR binary complex, 16 independent replicates were performed, with simulation times ranging from 500 ns to 6.4 μs. For the LM189-bound β$_2$AR binary complex, 10 independent replicates were conducted, with simulation times ranging from 600 ns to 11 μs.

CW-EPR spectroscopy

β2Δ5 with the Q142C mutation was expressed, purified, and labeled as described above. Frozen SEC pure receptor aliquots in 20 mM Hepes, pH 7.4, 100 mM NaCl, and 0.01% LMNG were thawed and incubated with ligands at 10× molar excess for 1 hour at RT; buffer matched or G protein was added at 2× molar excess to aliquots after 1-hour ligand incubation and incubated for another 2 hours. Apyrase (1 unit, NEB) was added for an additional 1.5 hours. Samples were then loaded into a quartz capillary [0.9 mm inner diameter (ID), 1.3 mm outer diameter (OD); #2-000-050, Drummond Scientific Company] with a volume of approximately 30 μl. CW-EPR spectroscopy was performed at X-band (~9.46 GHz) on a Bruker Magnettech ESR5000 spectrometer at RT. Spectra were recorded at a microwave power of 36 mW with 100-kHz field modulation at a modulation amplitude of 0.1 mT, a scan width of 20 mT, and a scan rate of 0.24 mT/s. CW data were aligned and baseline corrected using the custom software programs Convert&Align101 and Baseline048 written in LabVIEW by C. Altenbach (University of California, Los Angeles) and freely available upon request. Processed CW-EPR data were plotted in GraphPad Prism 9.3.1.

DEER spectroscopy

DEER samples consisted in approximately 100 μM spin-labeled β2Δ6 N148C/L266C, 10× molar excess of ligand, 2× molar excess of G proteins, or matched deuterated buffer. After 2 hours of incubation with the G protein, apyrase (1 unit NEB) was added for an additional 1.5 hours. Last, glycerol-d$_8$ was added as a cryoprotectant to a final concentration of 20% (v/v). Samples were loaded into borosilicate capillaries (1.4 mm ID, 1.7 mm OD; VitroCom) at final volumes of 14 to 20 μl and flash frozen in liquid nitrogen.

Experiments were performed at Q-band (~33.65 GHz) on a Bruker Elexsys E580 equipped with a SpinJet AWG, EN5107D2 resonator, variable-temperature cryogen-free cooling system (ColdEdge Technologies Inc.), and either a 10-W solid-state amplifier or a 150-W TWT amplifier (Applied Systems Engineering Inc.). The 150-W TWT amplifier was used to improve signal-to-noise ratio with respect to modulation for the transducer-coupled samples; data for all other samples were collected using the 10-W amplifier. Data were collected at a temperature of 50 K.

Dipolar evolution data were acquired using the dead-time free 4-pulse DEER sequence $(\pi/2)_{obs}$–(d_1)–$(\pi)_{obs}$–$(d_1 + T)$–$(\pi)_{pump}$–$(d_2 - T)$–$(\pi)_{obs}$–(d_2)–echo (*89*, *90*) with 16-step phase cycling (*91*). The parameters used for DEER experiments are listed in table S3. Gaussian shapes were implemented for all pulses using the built-in Gaussian pulse profile in Bruker Xepr software (*92*); resonator bandwidth compensation was not used. Pump pulses were applied to the maximal intensity of the field swept echo detected absorption spectrum. Observed pulses were applied at a frequency of either 45 or 90 MHz lower than the pump pulses for experiments performed with the 10- or 150-W amplifier, respectively. Optimal microwave power for π/2 and π pulses was determined by adjusting pulse amplitudes for a transient nutation experiment to achieve maximum Hahn echo inversion at the pulse lengths being used (72 ns) (*93*).

DEER data were processed with ComparativeDeerAnalyzer automated processing in DeerAnalysis2021 (*94*). This uses two different fitting routines: neural network analysis with DEERNet (*95*) from Spinach revision 5501 and Tikhonov regularization with DeerLab 0.9.1 routines (*96*). The resulting consensus fit is a mean of the two with a 95% confidence interval reported that is composed of both method's errors. DEER time traces and distance distributions for all samples are shown in fig. S5E; time traces are normalized to the signal intensity at time = 0 and distance distributions are area normalized. DEER data were plotted in GraphPad Prism 9.3.1.

smFRET spectroscopy

smPull receptor isolation and surface display

To inhibit nonspecific protein adsorption, flow cells for single-molecule experiments were prepared as previously described (*49*) using mPEG (Laysan Bio) passivated glass coverslips (VWR) and doped with biotin PEG16. Before each experiment, coverslips were incubated with NeutrAvidin (Thermo Fisher Scientific), followed by 10 nM biotinylated antibody (mouse anti-FLAG, Jackson ImmunoResearch). Between each conjugation step, the chambers were flushed to remove free reagents. The antibody dilutions and washes were done in T50 buffer (50 mM NaCl and 10 mM tris, pH 7.5). To achieve sparse immobilization of labeled receptors on the surface, purified labeled receptor was diluted (ranging from 100× to 1000× dilution) and applied to coverslips. After achieving optimum surface immobilization (~400 molecules in a 2000-μm^2 imaging area), unbound receptors were washed out of the flow chamber and the flow cells were then washed extensively (up to 50× the cell volume).

smFRET measurements

Receptors were imaged for smFRET in imaging buffer consisting of 3 mM Trolox, 100 mM NaCl, 2 mM $CaCl_2$, 20 mM Hepes, 0.01% MNG, and an oxygen scavenging system (0.8% dextrose, 0.8 mg ml^{-1} glucose oxidase, and 0.02 mg ml^{-1} catalase), pH 7.4. All buffers were made in UltraPure distilled water (Invitrogen). Samples were imaged with a 1.65 numerical aperture ×60 objective (Olympus) on a total internal reflection fluorescence microscope with 100 ms time resolution unless stated otherwise. Lasers at 532 nm (Cobolt) and 633 nm (Melles Griot) were used for donor and acceptor excitation, respectively. Fluorescence passed through Chroma ET550lp and split into donor and acceptor signal with a Chroma T635lpxr. FRET efficiency was calculated as $(I_A - 0.1I_D)/(I_D + I_A)$, in which I_D and I_A are the donor and acceptor intensity, respectively, after background subtraction. Movies were recorded at 100 ms acquisition time (10 Hz) with a Photometrics Prime 95B cMOS camera using micromanager acquisition software.

smFRET data analysis

SPARTAN version 3.7 (*97*) was used to analyze fluorescent movies. Donor and acceptor channels were aligned using the first 10 frames of each movie while excluding particles closer than 3.5 pixels and using an integration window of 12 pixels. Single-molecule intensity traces showing single-donor and single-acceptor photobleaching with a stable total intensity for longer than 5 s (50 frames), SNRbg >15, and donor acceptor correlation coefficient < 0.0 were collected (20 to 30% of total molecules per imaging area). Individual traces were smoothed using a nonlinear filter (*98*) with the following filter parameters: window = 2, $M = 2$, and $P = 15$. Each experiment was performed ≥4 times to ensure reproducibility. smFRET histograms

were compiled from ≥100 molecules per condition (100 ms time resolution). Error bars in the histograms represent the standard error from ≥4 independent movies. To ensure that traces of different lengths contribute equally, histograms from individual traces were normalized to one before compiling. Gaussian fitting to histograms was done in Origin Pro.

Fluorescence spectroscopy

Fluorescence experiments were performed on a Fluoromax 4C spectrofluorometer (Horiba Scientific, Edison, NJ, USA) using a 5-nm excitation slit width and a 3-nm emission slit width. Emission spectra were recorded using excitation at 380 nm. Concentrations after mixing were as follows: β_2AR, 100 nM; salmeterol, 100 μM; LM189, 75 μM; BI-167107, 25 μM. Ligand concentrations were chosen to achieve receptor saturation. Experiments were conducted in buffer containing 20 mM Hepes, pH 7.4, 100 mM NaCl, and 0.01% LMNG. Samples were measured after 1 hour incubation in the dark at the final concentrations to allow full equilibration. Measurements were performed in biological triplicates.

Gi and Gs coupling in intact HEK cells

HEK-A cells (or HEK-ΔGNAS) were cotransfected with rLuc-tagged β_2AR (β_2AR-rLuc8) and the Venus-miniGs (venus mGs) sensor containing the C-terminal residues from either $G\alpha_s$ or $G\alpha_{i1}$ (*11*, *99*, *100*) and a nuclear export signal (NES-venus-mGs) and allowed to express for 48 hours at 37°C in Dulbecco's modified Eagle's medium in a CO_2 incubator. These chimeras will be referred to as mGs and mGs/i. Expression vectors containing β_2AR-rLuc8 and the mGs chimeras were generously provided by Nevin Lambert (Augusta University at Georgia). Transfected cells were harvested with EDTA (2 mM) in PBS, washed (by centrifugation) in a Hepes buffered saline solution (HBSS), and resuspended in HBSS containing 0.1% ascorbic acid and 1% DMSO. Cells were transferred into 96-well plates (100 ml per well) and incubated for 20 min at RT. Cells were then preincubated with coelenterazine (5 mM final) for 5 min before the addition of agonist. An agonist dose-response relationship was performed through the addition of a 10× agonist concentration to the cells, and the fluorescence emission was measured (at 485 and 530 nm) using a Molecular Devices M5 fluorescence plate reader. The data were collected in kinetic mode every 120 s for 30 min total. Activity values were derived from the area under the BRET ratio (em530/em485) progress curve between 6 and 16 min. Data were analyzed using Prism (GraphPad, La Jolla, CA).

Supplementary Materials

This PDF file includes:
Figs. S1 to S6
Tables S1 to S3
Legends for data S1 and S2

Other Supplementary Material for this manuscript includes the following:
Data S1 and S2

REFERENCES AND NOTES

1. R. Fredriksson, M. C. Lagerström, L.-G. Lundin, H. B. Schiöth, The G-protein-coupled receptors in the human genome form five main families. Phylogenetic analysis, paralogon groups, and fingerprints. *Mol. Pharmacol.* **63**, 1256–1272 (2003).
2. I. Masuho, O. Ostrovskaya, G. M. Kramer, C. D. Jones, K. Xie, K. A. Martemyanov, Distinct profiles of functional discrimination among G proteins determine the actions of G protein–coupled receptors. *Sci. Signal.* **8**, ra123 (2015).
3. A. Koehl, H. Hu, S. Maeda, Y. Zhang, Q. Qu, J. M. Paggi, N. R. Latorraca, D. Hilger, R. Dawson, H. Matile, G. F. X. Schertler, S. Granier, W. I. Weis, R. O. Dror, A. Manglik, G. Skiniotis, B. K. Kobilka, Structure of the μ-opioid receptor–Gi protein complex. *Nature* **558**, 547–552 (2018).
4. S. G. F. Rasmussen, B. T. DeVree, Y. Zou, A. C. Kruse, K. Y. Chung, T. S. Kobilka, F. S. Thian, P. S. Chae, E. Pardon, D. Calinski, J. M. Mathiesen, S. T. A. Shah, J. A. Lyons, M. Caffrey, S. H. Gellman, J. Steyaert, G. Skiniotis, W. I. Weis, R. K. Sunahara, B. K. Kobilka, Crystal structure of the β2 adrenergic receptor–Gs protein complex. *Nature* **477**, 549–555 (2011).
5. K. Krishna Kumar, M. Shalev-Benami, M. J. Robertson, H. Hu, S. D. Banister, S. A. Hollingsworth, N. R. Latorraca, H. E. Kato, D. Hilger, S. Maeda, W. I. Weis, D. L. Farrens, R. O. Dror, S. V. Malhotra, B. K. Kobilka, G. Skiniotis, Structure of a signaling cannabinoid receptor 1-G protein complex. *Cell* **176**, 448–458.e12 (2019).
6. S. Maeda, Q. Qu, M. J. Robertson, G. Skiniotis, B. K. Kobilka, Structures of the M1 and M2 muscarinic acetylcholine receptor/G-protein complexes. *Science* **364**, 552–557 (2019).
7. Y. Zhuang, P. Xu, C. Mao, L. Wang, B. Krumm, X. E. Zhou, S. Huang, H. Liu, X. Cheng, X.-P. Huang, D.-D. Shen, T. Xu, Y.-F. Liu, Y. Wang, J. Guo, Y. Jiang, H. Jiang, K. Melcher, B. L. Roth, Y. Zhang, C. Zhang, H. E. Xu, Structural insights into the human D1 and D2 dopamine receptor signaling complexes. *Cell* **184**, 931–942.e18 (2021).
8. S. Huang, P. Xu, D.-D. Shen, I. A. Simon, C. Mao, Y. Tan, H. Zhang, K. Harpsøe, H. Li, Y. Zhang, C. You, X. Yu, Y. Jiang, Y. Zhang, D. E. Gloriam, H. E. Xu, GPCRs steer Gi and Gs selectivity via TM5-TM6 switches as revealed by structures of serotonin receptors. *Mol. Cell* **82**, 2681–2695.e6 (2022).
9. A. Qiao, S. Han, X. Li, Z. Li, P. Zhao, A. Dai, R. Chang, L. Tai, Q. Tan, X. Chu, L. Ma, T. S. Thorsen, S. Reedtz-Runge, D. Yang, M.-W. Wang, P. M. Sexton, D. Wootten, F. Sun, Q. Zhao, B. Wu, Structural basis of G_s and G_i recognition by the human glucagon receptor. *Science* **367**, 1346–1352 (2020).
10. T. Flock, A. S. Hauser, N. Lund, D. E. Gloriam, S. Balaji, M. M. Babu, Selectivity determinants of GPCR–G-protein binding. *Nature* **545**, 317–322 (2017).
11. N. Okashah, Q. Wan, S. Ghosh, M. Sandhu, A. Inoue, N. Vaidehi, N. A. Lambert, Variable G protein determinants of GPCR coupling selectivity. *Proc. Natl. Acad. Sci. U.S.A.* **116**, 12054–12059 (2019).
12. A. Inoue, F. Raimondi, F. M. N. Kadji, G. Singh, T. Kishi, A. Uwamizu, Y. Ono, Y. Shinjo, S. Ishida, N. Arang, K. Kawakami, J. S. Gutkind, J. Aoki, R. B. Russell, Illuminating G-protein-coupling selectivity of GPCRs. *Cell* **177**, 1933–1947.e25 (2019).
13. H. R. Kim, J. Xu, S. Maeda, N. M. Duc, D. Ahn, Y. Du, K. Y. Chung, Structural mechanism underlying primary and secondary coupling between GPCRs and the Gi/o family. *Nat. Commun.* **11**, 3160 (2020).
14. X. Ma, Y. Hu, H. Batebi, J. Heng, J. Xu, X. Liu, X. Niu, H. Li, P. W. Hildebrand, C. Jin, B. K. Kobilka, Analysis of β_2AR-G_s and β_2AR-G_i complex formation by NMR spectroscopy. *Proc. Natl. Acad. Sci. U.S.A.* **117**, 23096–23105 (2020).
15. F. Horn, E. M. van der Wenden, L. Oliveira, A. P. IJzerman, G. Vriend, Receptors coupling to G proteins: Is there a signal behind the sequence? *Proteins* **41**, 448–459 (2000).
16. G. J. Rodriguez, R. Yao, O. Lichtarge, T. G. Wensel, Evolution-guided discovery and recoding of allosteric pathway specificity determinants in psychoactive bioamine receptors. *Proc. Natl. Acad. Sci. U.S.A.* **107**, 7787–7792 (2010).
17. A. Manglik, T. H. Kim, M. Masureel, C. Altenbach, Z. Yang, D. Hilger, M. T. Lerch, T. S. Kobilka, F. S. Thian, W. L. Hubbell, R. S. Prosser, B. K. Kobilka, Structural insights into the dynamic process of β2-adrenergic receptor signaling. *Cell* **161**, 1101–1111 (2015).
18. R. Nygaard, Y. Zou, R. O. Dror, T. J. Mildorf, D. H. Arlow, A. Manglik, A. C. Pan, C. W. Liu, J. J. Fung, M. P. Bokoch, F. S. Thian, T. S. Kobilka, D. E. Shaw, L. Mueller, R. S. Prosser, B. K. Kobilka, The dynamic process of β2-adrenergic receptor activation. *Cell* **152**, 532–542 (2013).
19. L. Ye, N. Van Eps, M. Zimmer, O. P. Ernst, R. S. Prosser, Activation of the A2A adenosine G-protein-coupled receptor by conformational selection. *Nature* **533**, 265–268 (2016).
20. X. Deupi, B. K. Kobilka, Energy landscapes as a tool to integrate GPCR structure, dynamics, and function. *Physiology* **25**, 293–303 (2010).
21. J. Okude, T. Ueda, Y. Kofuku, M. Sato, N. Nobuyama, K. Kondo, Y. Shiraishi, T. Mizumura, K. Onishi, M. Natsume, M. Maeda, H. Tsujishita, T. Kuranaga, M. Inoue, I. Shimada, Identification of a conformational equilibrium that determines the efficacy and functional selectivity of the μ-opioid receptor. *Angew. Chem. Int. Ed. Engl.* **54**, 15771–15776 (2015).
22. L. M. Wingler, M. Elgeti, D. Hilger, N. R. Latorraca, M. T. Lerch, D. P. Staus, R. O. Dror, B. K. Kobilka, W. L. Hubbell, R. J. Lefkowitz, Analogs with divergent bias stabilize distinct receptor conformations. *Cell* **176**, 468–478.e11 (2019).
23. J. J. Liu, R. Horst, V. Katritch, R. C. Stevens, K. Wüthrich, Biased signaling pathways in β_2-adrenergic receptor characterized by ^{19}F-NMR. *Science* **335**, 1106–1110 (2012).
24. R. Rahmeh, M. Damian, M. Cottet, H. Orcel, C. Mendre, T. Durroux, K. S. Sharma, G. Durand, B. Pucci, E. Trinquet, J. M. Zwier, X. Deupi, P. Bron, J.-L. Banères, B. Mouillac, S. Granier, Structural insights into biased G protein-coupled receptor signaling revealed by fluorescence spectroscopy. *Proc. Natl. Acad. Sci. U.S.A.* **109**, 6733–6738 (2012).
25. J. Wang, K. Hanada, D. P. Staus, M. A. Makara, G. R. Dahal, Q. Chen, A. Ahles, S. Engelhardt, H. A. Rockman, $G\alpha_i$ is required for carvedilol-induced β1 adrenergic receptor β-arrestin biased signaling. *Nat. Commun.* **8**, 1706 (2017).
26. A. Y.-H. Woo, Y. Song, R.-P. Xiao, W. Zhu, Biased β_2-adrenoceptor signalling in heart failure: Pathophysiology and drug discovery. *Br. J. Pharmacol.* **172**, 5444–5456 (2015).

27. A. Chesley, M. S. Lundberg, T. Asai, R.-P. Xiao, S. Ohtani, E. G. Lakatta, M. T. Crow, The β_2-adrenergic receptor delivers an antiapoptotic signal to cardiac myocytes through G_i-dependent coupling to phosphatidylinositol 3′-kinase. *Circ. Res.* **87**, 1172–1179 (2000).
28. W. Z. Zhu, M. Zheng, W. J. Koch, R. J. Lefkowitz, B. K. Kobilka, R. P. Xiao, Dual modulation of cell survival and cell death by β_2-adrenergic signaling in adult mouse cardiac myocytes. *Proc. Natl. Acad. Sci. U.S.A.* **98**, 1607–1612 (2001).
29. G. Fajardo, M. Zhao, T. Urashima, S. Farahani, D.-Q. Hu, S. Reddy, D. Bernstein, Deletion of the β2-adrenergic receptor prevents the development of cardiomyopathy in mice. *J. Mol. Cell. Cardiol.* **63**, 155–164 (2013).
30. Y. Kofuku, T. Ueda, J. Okude, Y. Shiraishi, K. Kondo, M. Maeda, H. Tsujishita, I. Shimada, Efficacy of the β_2-adrenergic receptor is determined by conformational equilibrium in the transmembrane region. *Nat. Commun.* **3**, 1045 (2012).
31. K. Y. Chung, S. G. F. Rasmussen, T. Liu, S. Li, B. T. DeVree, P. S. Chae, D. Calinski, B. K. Kobilka, V. L. Woods, R. K. Sunahara, Conformational changes in the G protein Gs induced by the β2 adrenergic receptor. *Nature* **477**, 611–615 (2011).
32. G. G. Gregorio, M. Masureel, D. Hilger, D. S. Terry, M. Juette, H. Zhao, Z. Zhou, J. M. Perez-Aguilar, M. Hauge, S. Mathiasen, J. A. Javitch, H. Weinstein, B. K. Kobilka, S. C. Blanchard, Single-molecule analysis of ligand efficacy in β_2AR–G-protein activation. *Nature* **547**, 68–73 (2017).
33. Y. Du, N. M. Duc, S. G. F. Rasmussen, D. Hilger, X. Kubiak, L. Wang, J. Bohon, H. R. Kim, M. Wegrecki, A. Asuru, K. M. Jeong, J. Lee, M. R. Chance, D. T. Lodowski, B. K. Kobilka, Y. ChungK, Assembly of a GPCR-G protein complex. *Cell* **3**, 1045 (2012).
34. Y. Daaka, L. M. Luttrell, R. J. Lefkowitz, Switching of the coupling of the β_2-adrenergic receptor to different G proteins by protein kinase A. *Nature* **390**, 88–91 (1997).
35. M. J. Strohman, S. Maeda, D. Hilger, M. Masureel, Y. Du, B. K. Kobilka, Local membrane charge regulates β2 adrenergic receptor coupling to G_{i3}. *Nat. Commun.* **10**, 2234 (2019).
36. E. Devic, Y. Xiang, D. Gould, B. Kobilka, β-adrenergic receptor subtype-specific signaling in cardiac myocytes from β_1 and β_2 adrenoceptor knockout mice. *Mol. Pharmacol.* **60**, 577–583 (2001).
37. J. I. Mobbs, M. J. Belousoff, K. G. Harikumar, S. J. Piper, X. Xu, S. G. B. Furness, H. Venugopal, A. Christopoulos, R. Danev, D. Wootten, D. M. Thal, L. J. Miller, P. M. Sexton, Structures of the human cholecystokinin 1 (CCK1) receptor bound to Gs and Gq mimetic proteins provide insight into mechanisms of G protein selectivity. *PLOS Biol.* **19**, e3001295 (2021).
38. M. Masureel, Y. Zou, L.-P. Picard, E. van der Westhuizen, J. P. Mahoney, J. P. G. L. M. Rodrigues, T. J. Mildorf, R. O. Dror, D. E. Shaw, M. Bouvier, E. Pardon, J. Steyaert, R. K. Sunahara, W. I. Weis, C. Zhang, B. K. Kobilka, Structural insights into binding specificity, efficacy and bias of a β_2AR partial agonist. *Nat. Chem. Biol.* **14**, 1059–1066 (2018).
39. M. Weatherall, M. Wijesinghe, K. Perrin, M. Harwood, R. Beasley, Meta-analysis of the risk of mortality with salmeterol and the effect of concomitant inhaled corticosteroid therapy. *Thorax* **65**, 39–43 (2010).
40. T. Kenakin, C. Watson, V. Muniz-Medina, A. Christopoulos, S. Novick, A simple method for quantifying functional selectivity and agonist bias. *ACS Chem. Nerosci.* **3**, 193–203 (2012).
41. E. T. van der Westhuizen, B. Breton, A. Christopoulos, M. Bouvier, Quantification of ligand bias for clinically relevant β_2-adrenergic receptor ligands: Implications for drug taxonomy. *Mol. Pharmacol.* **85**, 492–509 (2014).
42. S. Ahn, B. Pani, A. W. Kahsai, E. K. Olsen, G. Husemoen, M. Vestergaard, L. Jin, S. Zhao, L. M. Wingler, P. K. Rambarat, R. K. Simhal, T. T. Xu, L. D. Sun, P. J. Shim, D. P. Staus, L.-Y. Huang, T. Franch, X. Chen, R. J. Lefkowitz, Small-molecule positive allosteric modulators of the β_2-adrenoceptor isolated from DNA-encoded libraries. *Mol. Pharmacol.* **94**, 850–861 (2018).
43. X. Liu, A. Masoudi, A. W. Kahsai, L.-Y. Huang, B. Pani, D. P. Staus, P. J. Shim, K. Hirata, R. K. Simhal, A. M. Schwalb, P. K. Rambarat, S. Ahn, R. J. Lefkowitz, B. Kobilka, Mechanism of β_2AR regulation by an intracellular positive allosteric modulator. *Science* **364**, 1283–1287 (2019).
44. V. Cherezov, D. M. Rosenbaum, M. A. Hanson, S. G. F. Rasmussen, F. S. Thian, T. S. Kobilka, H.-J. Choi, P. Kuhn, W. I. Weis, B. K. Kobilka, R. C. Stevens, High-resolution crystal structure of an engineered human β_2-adrenergic G protein–coupled receptor. *Science* **318**, 1258–1265 (2007).
45. C. Altenbach, C. J. López, K. Hideg, W. L. Hubbell, Chapter three—Exploring structure, dynamics, and topology of nitroxide spin-labeled proteins using continuous-wave electron paramagnetic resonance spectroscopy. *Methods Enzymol.* **564**, 59–100 (2015).
46. G. Peleg, P. Ghanouni, B. K. Kobilka, R. N. Zare, Single-molecule spectroscopy of the β_2 adrenergic receptor: Observation of conformational substates in a membrane protein. *Proc. Natl. Acad. Sci. U.S.A.* **98**, 8469–8474 (2001).
47. P. Ghanouni, Z. Gryczynski, J. J. Steenhuis, T. W. Lee, D. L. Farrens, J. R. Lakowicz, B. K. Kobilka, Functionally different agonists induce distinct conformations in the G protein coupling domain of the β2 adrenergic receptor. *J. Biol. Chem.* **276**, 24433–24436 (2001).
48. C. H. Habrian, J. Levitz, V. Vyklicky, Z. Fu, A. Hoagland, I. McCort-Tranchepain, F. Acher, E. Y. Isacoff, Conformational pathway provides unique sensitivity to a synaptic mGluR. *Nat. Commun.* **10**, 5572 (2019).
49. R. Vafabakhsh, J. Levitz, E. Y. Isacoff, Conformational dynamics of a class C G-protein-coupled receptor. *Nature* **524**, 497–501 (2015).
50. J. García-Nafría, C. G. Tate, Cryo-EM structures of GPCRs coupled to G_s, G_i and G_o. *Mol. Cell. Endocrinol.* **488**, 1–13 (2019).
51. M. M. Papasergi-Scott, G. Pérez-Hernández, H. Batebi, Y. Gao, G. Eskici, A. B. Seven, O. Panova, D. Hilger, M. Casiraghi, F. He, L. Maul, P. Gmeiner, B. K. Kobilka, P. W. Hildebrand, G. Skiniotis, Time-resolved cryo-EM of G-protein activation by a GPCR. *Nature* **629**, 1182–1191 (2024).
52. M. Choi, D. P. Staus, L. M. Wingler, S. Ahn, B. Pani, W. D. Capel, R. J. Lefkowitz, G protein–coupled receptor kinases (GRKs) orchestrate biased agonism at the β_2-adrenergic receptor. *Sci. Signal.* **11**, eaar7084 (2018).
53. I. F. Skidmore, A. Naylor, L. H. C. Lunts, H. Finch, Ethanolamine compounds (1985).
54. I. F. Skidmore A. Naylor, H. Finch, L. H. C. Lunts, I. B. Campbell, Phenethanolamine derivatives (1988).
55. X. Xu, J. Shonberg, J. Kaindl, M. J. Clark, A. Stößel, L. Maul, D. Mayer, H. Hübner, K. Hirata, A. J. Venkatakrishnan, R. O. Dror, B. K. Kobilka, R. K. Sunahara, X. Liu, P. Gmeiner, Constrained catecholamines gain β_2AR selectivity through allosteric effects on pocket dynamics. *Nat. Commun.* **14**, 2138 (2023).
56. M. Stanek, L.-P. Picard, M. F. Schmidt, J. M. Kaindl, H. Hübner, M. Bouvier, D. Weikert, P. Gmeiner, Hybridization of β-adrenergic agonists and antagonists confers G protein bias. *J. Med. Chem.* **62**, 5111–5131 (2019).
57. O. H. Lowry, N. J. Rosebrough, A. L. Farr, R. J. Randall, Protein measurement with the Folin phenol reagent. *J. Biol. Chem.* **193**, 265–275 (1951).
58. Y.-C. Cheng, W. H. Prusoff, Relationship between the inhibition constant (K_i) and the concentration of inhibitor which causes 50 per cent inhibition (I_{50}) of an enzymatic reaction. *Biochem. Pharmacol.* **22**, 3099–3108 (1973).
59. X. Liu, J. Kaindl, M. Korczynska, A. Stößel, D. Dengler, M. Stanek, H. Hübner, M. J. Clark, J. Mahoney, R. A. Matt, X. Xu, K. Hirata, B. K. Shoichet, R. K. Sunahara, B. K. Kobilka, P. Gmeiner, An allosteric modulator binds to a conformational hub in the β2 adrenergic receptor. *Nat. Chem. Biol.* **16**, 749–755 (2020).
60. J. Zivanov, T. Nakane, B. O. Forsberg, D. Kimanius, W. J. Hagen, E. Lindahl, S. H. W. Scheres, New tools for automated high-resolution cryo-EM structure determination in RELION-3. *eLife* **7**, e42166 (2018).
61. A. Rohou, N. Grigorieff, CTFFIND4: Fast and accurate defocus estimation from electron micrographs. *J. Struct. Biol.* **192**, 216–221 (2015).
62. A. Punjani, H. Zhang, D. J. Fleet, Non-uniform refinement: Adaptive regularization improves single-particle cryo-EM reconstruction. *Nat. Methods* **17**, 1214–1221 (2020).
63. Y. Z. Tan, P. R. Baldwin, J. H. Davis, J. R. Williamson, C. S. Potter, B. Carragher, D. Lyumkis, Addressing preferred specimen orientation in single-particle cryo-EM through tilting. *Nat. Methods* **14**, 793–796 (2017).
64. N. W. Moriarty, R. W. Grosse-Kunstleve, P. D. Adams, Electronic Ligand Builder and Optimization Workbench (eLBOW): A tool for ligand coordinate and restraint generation. *Acta Crystallogr. D Biol. Crystallogr.* **65**, 1074–1080 (2009).
65. M. Manna, M. Niemelä, J. Tynkkynen, M. Javanainen, W. Kulig, D. J. Müller, T. Rog, I. Vattulainen, Mechanism of allosteric regulation of β_2-adrenergic receptor by cholesterol. *eLife* **5**, e18432 (2016).
66. A. Casañal, B. Lohkamp, P. Emsley, Current developments in *Coot* for macromolecular model building of electron cryo-microscopy and crystallographic data. *Protein Sci.* **29**, 1055–1064 (2020).
67. A. M. Ring, A. Manglik, A. C. Kruse, M. D. Enos, W. I. Weis, K. C. Garcia, B. K. Kobilka, Adrenaline-activated structure of β2-adrenoceptor stabilized by an engineered nanobody. *Nature* **502**, 575–579 (2013).
68. E. F. Pettersen, T. D. Goddard, C. C. Huang, G. S. Couch, D. M. Greenblatt, E. C. Meng, T. E. Ferrin, UCSF Chimera—A visualization system for exploratory research and analysis. *J. Comput. Chem.* **25**, 1605–1612 (2004).
69. B. Webb, A. Sali, Comparative protein structure modeling using MODELLER. *Curr. Protoc. Bioinformatics* **54**, 5.6.1–5.6.37 (2016).
70. A. Ranganathan, R. O. Dror, J. Carlsson, Insights into the role of Asp79$^{2.50}$ in β_2 adrenergic receptor activation from molecular dynamics simulations. *Biochemistry.* **53**, 7283–7296 (2014).
71. R. O. Dror, D. H. Arlow, D. W. Borhani, M. Ø. Jensen, S. Piana, D. E. Shaw, Identification of two distinct inactive conformations of the β_2 -adrenergic receptor reconciles structural and biochemical observations. *Proc. Natl. Acad. Sci. U.S.A.* **106**, 4689–4694 (2009).
72. D. M. Rosenbaum, C. Zhang, J. A. Lyons, R. Holl, D. Aragao, D. H. Arlow, S. G. F. Rasmussen, H.-J. Choi, B. T. DeVree, R. K. Sunahara, P. S. Chae, S. H. Gellman, R. O. Dror, D. E. Shaw, W. I. Weis, M. Caffrey, P. Gmeiner, B. K. Kobilka, Structure and function of an irreversible agonist-β2 adrenoceptor complex. *Nature* **469**, 236–240 (2011).
73. D. A. Case, I. Y. Ben-Shalom, S. R. Brozell, D. S. Cerutti, T. E. Cheatham III, V. W. D. Cruzeiro, T. A. Darden, R. E. Duke, D. Ghoreishi, M. K. Gilson, H. Gohlke, A. W. Goetz, D. Greene, R. Harris, N. Homeyer, S. Izadi, A. Kovalenko, T. Kurtzman, T. S. Lee, S. LeGrand, P. Li, C. Lin, J. Liu, T. Luchko, R. Luo, D. J. Mermelstein, K. M. Merz, Y. Miao, G. Monard, C. Nguyen, H. Nguyen, I. Omelyan, A. Onufriev, F. Pan, R. Qi, D. R. Roe, A. Roitberg, C. Sagui, S. Schott-Verdugo, J. Shen, C. L. Simmerling, J. Smith, R. Salomon-Ferrer, J. Swails,

R. C. Walker, J. Wang, H. Wei, R. M. Wolf, X. Wu, L. Xiao, D. M. York, P. A. Kollman, *AMBER 2018* (University of California, San Francisco, 2018).
74. M. J. Frisch, G. W. Trucks, H. B. Schlegel, G. E. Scuseria, M. A. Robb, J. R. Cheeseman, G. Scalmani, V. Barone, G. A. Petersson, H. Nakatsuji, X. Li, M. Caricato, A. V. Marenich, J. Bloino, B. G. Janesko, R. Gomperts, B. Mennucci, H. P. Hratchian, J. V. Ortiz, A. F. Izmaylov, J. L. Sonnenberg, D. Williams-Young, F. Ding, F. Lipparini, F. Egidi, J. Goings, B. Peng, A. Petrone, T. Henderson, D. Ranasinghe, V. G. Zakrzewski, J. Gao, N. Rega, G. Zheng, W. Liang, M. Hada, M. Ehara, K. Toyota, R. Fukuda, J. Hasegawa, M. Ishida, T. Nakajima, Y. Honda, O. Kitao, H. Nakai, T. Vreven, K. Throssell, J. A. Montgomery, Jr., J. E. Peralta, F. Ogliaro, M. J. Bearpark, J. J. Heyd, E. N. Brothers, K. N. Kudin, V. N. Staroverov, T. A. Keith, R. Kobayashi, J. Normand, K. Raghavachari, A. P. Rendell, J. C. Burant, S. S. Iyengar, J. Tomasi, M. Cossi, J. M. Millam, M. Klene, C. Adamo, R. Cammi, J. W. Ochterski, R. L. Martin, K. Morokuma, O. Farkas, J. B. Foresman, D. J. Fox, *Gaussian 16, version Revision C.01* (Gaussian Inc., 2016).
75. C. I. Bayly, P. Cieplak, W. Cornell, P. A. Kollman, A well-behaved electrostatic potential based method using charge restraints for deriving atomic charges: The RESP model. *J. Phys. Chem.* **97**, 10269–10280 (1993).
76. M. A. Lomize, A. L. Lomize, I. D. Pogozheva, H. I. Mosberg, OPM: Orientations of Proteins in Membranes database. *Bioinformatics* **22**, 623–625 (2006).
77. M. G. Wolf, M. Hoefling, C. Aponte-Santamaría, H. Grubmüller, G. Groenhof, g_membed: Efficient insertion of a membrane protein into an equilibrated lipid bilayer with minimal perturbation. *J. Comput. Chem.* **31**, 2169–2174 (2010).
78. J. Wang, R. M. Wolf, J. W. Caldwell, P. A. Kollman, D. A. Case, Development and testing of a general amber force field. *J. Comput. Chem.* **25**, 1157–1174 (2004).
79. C. J. Dickson, B. D. Madej, Å. A. Skjevik, R. M. Betz, K. Teigen, I. R. Gould, R. C. Walker, Lipid14: The amber lipid force field. *J. Chem. Theory Comput.* **10**, 865–879 (2014).
80. J. A. Maier, C. Martinez, K. Kasavajhala, L. Wickstrom, K. E. Hauser, C. Simmerling, ff14SB: Improving the accuracy of protein side chain and backbone parameters from ff99SB. *J. Chem. Theory Comput.* **11**, 3696–3713 (2015).
81. H. J. C. Berendsen, J. R. Grigera, T. P. Straatsma, The missing term in effective pair potentials. *J. Phys. Chem.* **91**, 6269–6271 (1987).
82. D. Van Der Spoel, E. Lindahl, B. Hess, G. Groenhof, A. E. Mark, H. J. C. Berendsen, GROMACS: Fast, flexible, and free. *J. Comput. Chem.* **26**, 1701–1718 (2005).
83. M. J. Abraham, T. Murtola, R. Schulz, S. Páll, J. C. Smith, B. Hess, E. Lindahl, GROMACS: High performance molecular simulations through multi-level parallelism from laptops to supercomputers. *SoftwareX* **1–2**, 19–25 (2015).
84. E. Lindahl, M. J. Abraham, B. Hess, V. D. Spoel, *GROMACS 2021 Manual* (2021).
85. B. Hess, H. Bekker, H. J. C. Berendsen, J. G. E. M. Fraaije, LINCS: A linear constraint solver for molecular simulations. *J. Comput. Chem.* **18**, 1463–1472 (1997).
86. T. Darden, D. York, L. Pedersen, Particle mesh Ewald: An $N\log(N)$ method for Ewald sums in large systems. *J. Chem. Phys.* **98**, 10089–10092 (1993).
87. D. R. Roe, T. E. Cheatham, PTRAJ and CPPTRAJ: Software for processing and analysis of molecular dynamics trajectory data. *J. Chem. Theory Comput.* **9**, 3084–3095 (2013).
88. J. D. Hunter, Matplotlib: A 2D graphics environment. *Comput. Sci. Eng.* **9**, 90–95 (2007).
89. I. Tkach, U. Diederichsen, M. Bennati, Studies of transmembrane peptides by pulse dipolar spectroscopy with semi-rigid TOPP spin labels. *Eur. Biophys. J.* **50**, 143–157 (2021).
90. B. Joseph, E. A. Jaumann, A. Sikora, K. Barth, T. F. Prisner, D. S. Cafiso, In situ observation of conformational dynamics and protein ligand–substrate interactions in outer-membrane proteins with DEER/PELDOR spectroscopy. *Nat. Protoc.* **14**, 2344–2369 (2019).
91. C. E. Tait, S. Stoll, Coherent pump pulses in double electron electron resonance spectroscopy. *Phys. Chem. Chem. Phys.* **18**, 18470–18485 (2016).
92. M. Teucher, E. Bordignon, Improved signal fidelity in 4-pulse DEER with Gaussian pulses. *J. Magn. Reson.* **296**, 103–111 (2018).
93. H. Russell, R. Stewart, C. Prior, V. S. Oganesyan, T. G. Gaule, J. E. Lovett, DEER and RIDME measurements of the nitroxide-spin labelled copper-bound amine oxidase homodimer from arthrobacter globiformis. *Appl Magn Reson* **52**, 995–1015 (2021).
94. O. Schiemann, C. A. Heubach, D. Abdullin, K. Ackermann, M. Azarkh, E. G. Bagryanskaya, M. Drescher, B. Endeward, J. H. Freed, L. Galazzo, D. Goldfarb, T. Hett, L. E. Hofer, L. F. Ibáñez, E. J. Hustedt, S. Kucher, I. Kuprov, J. E. Lovett, A. Meyer, S. Ruthstein, S. Saxena, S. Stoll, C. R. Timmel, M. Di Valentin, H. S. Mchaourab, T. F. Prisner, B. E. Bode, E. Bordignon, M. Bennati, G. Jeschke, Benchmark test and guidelines for DEER/PELDOR experiments on nitroxide-labeled biomolecules. *J. Am. Chem. Soc.* **143**, 17875–17890 (2021).
95. J. Keeley, T. Choudhury, L. Galazzo, E. Bordignon, A. Feintuch, D. Goldfarb, H. Russell, M. J. Taylor, J. E. Lovett, A. Eggeling, L. Fábregas Ibáñez, K. Keller, M. Yulikov, G. Jeschke, I. Kuprov, Neural networks in pulsed dipolar spectroscopy: A practical guide. *J. Magn. Reson.* **338**, 107186 (2022).
96. L. Fábregas Ibáñez, G. Jeschke, S. Stoll, DeerLab: A comprehensive software package for analyzing dipolar electron paramagnetic resonance spectroscopy data. *Magn. Reson.* **1**, 209–224 (2020).
97. M. F. Juette, D. S. Terry, M. R. Wasserman, R. B. Altman, Z. Zhou, H. Zhao, S. C. Blanchard, Single-molecule imaging of non-equilibrium molecular ensembles on the millisecond timescale. *Nat. Methods.* **13**, 341–344 (2016).
98. G. Haran, Noise reduction in single-molecule fluorescence trajectories of folding proteins. *Chem. Phys.* **307**, 137–145 (2004).
99. Q. Wan, N. Okashah, A. Inoue, R. Nehmé, B. Carpenter, C. G. Tate, N. A. Lambert, Mini G protein probes for active G protein–coupled receptors (GPCRs) in live cells. *J. Biol. Chem.* **293**, 7466–7473 (2018).
100. R. Nehmé, B. Carpenter, A. Singhal, A. Strege, P. C. Edwards, C. F. White, H. Du, R. Grisshammer, C. G. Tate, Mini-G proteins: Novel tools for studying GPCRs in their active conformation. *PLOS ONE* **12**, e0175642 (2017).

Acknowledgments: We thank E. Montabana for assistance with the cryo-EM data collection. We acknowledge A. W. Kahsai for cmpd-6FA. We acknowledge National Institutes of Health Instrumentation grant S10OD024980 in support of acquisition of the UC Santa Cruz pulsed EPR spectrometer. We thank K. Sato, S. Nakano, and A. Inoue (Tohoku University) for their assistance in the NanoBiT assay. We thank N. Lambert (Augusta University) for the β_2AR-rLuc8 and miniG constructs as well as helpful discussion. Cryo-EM data were collected at Stanford Cryo-Electron Microscopy Center (cEMc). The materials used in this study can be provided by B.K.K., M.T.L., and P.G. pending scientific review and a completed material transfer agreement. Requests for the materials should be submitted to M.C. (mcasirag@stanford.edu), B.K.K., M.T.L., and P.G. **Funding:** M.C. received funding from the European Union Horizon 2020 research and innovation program under the Marie Sklodowska-Curie grant agreement no. 799376. M.C. was supported by the American Heart Association (AHA) Postdoctoral Fellowship award number 915188. B.K.K. was supported by R01NS028471. M.T.L. is supported by R01GM135581 and grant S10 OD025260. P.G. is supported by the Deutsche Forschungsgemeinschaft (DFG, German Research Foundation) grants GRK 1910 and GM 13/14-1. R.J.L. is an investigator with the Howard Hughes Medical Institute and is supported by R01HL0160371609 from the National Heart, Lung, and Blood Institute, NIH. P.C.B. is supported by F31 HL164002. E.Y.I. is a Weill Neurohub Investigator and is supported by R01NS119826. N.N. was supported by NSF MCB-1942957 and American Chemical Society Petroleum Research Fund, 61678-UR6. Y.K.X. is supported by NIH grant HL162825 and MH134119 and VA Merit grants IK6BX005753 and I01BX005100. A.I. is funded by the Society for the Promotion of Science (JP21H04791 and JP24K2128), the Japan Science and Technology Agency (JPMJFR215T and JPMJMS2023), and the Japan Agency for Medical Research and Development (JP22ama121038 and JP22zf0127007). R.K.S. is funded by National Institute of General Medical Sciences grant GM083118. **Author contributions:** Conceptualization: M.C., B.K.K., M.T.L., H.W., R.J.L., B.P., and P.C.B. Methodology: M.C., B.K.K., M.T.L., P.G., R.J.L., N.N., E.Y.I., A.I., R.K.S., H.W., B.P., P.C.B., L.M., and S.M.F.M.B. Software: H.W. Validation: M.C., B.K.K., P.G., P.C.B., R.K.S., N.N., and S.M.F.M.B. Formal analysis: M.C., P.C.B., R.K.S., N.N., C.H., L.M., S.M.F.M.B., and Y.K.X. Investigation: M.C., H.W., P.C.B., N.N., H.H., L.M., M.F.S., S.M.F.M.B., B.X., R.K.S., T.E.A., E.W., A.I., T.E.A., N.S., A.I., and B.C. Resources: B.K.K., P.G., M.T.L., M.C., B.P., P.C.B., L.M., Y.K.X., and H.W. Data curation: N.N. and H.W. Visualization: M.C., H.W., P.C.B., C.H., H.H., M.F.S., A.I., N.S., R.J.L., E.Y.I., N.N., S.M.F.M.B., and Y.K.X. Funding acquisition: M.C., B.K.K., M.T.L., P.C.B., P.G., R.J.L., E.Y.I., N.N., Y.K.X., A.I., and R.K.S. Project administration: M.C., B.K.K., M.T.L., P.G., and R.J.L. Supervision: B.K.K., P.G., M.T.L., M.C., R.J.L., E.Y.I., and Y.K.X. Writing—original draft: M.C., B.K.K., H.W., and P.C.B. Writing—review and editing: M.C., B.K.K., M.T.L., P.G., R.J.L., N.N., E.Y.I., P.C.B., B.P., A.I., N.S., L.M., M.F.S., A.I., B.C., and S.M.F.M.B. **Competing interests:** B.K.K. is a cofounder of and consultant for ConfometRx Inc. R.J.L. is a founder of Trevena Inc. and a cofounder and stockholder of Septerna. R.J.L. is also a cofounder and stockholder of Lexicon Pharmaceuticals. B.P. is a consultant with Septerna. All other authors declare that they have no competing interests. **Data and materials availability:** The cryo-EM density map has been deposited in the Electron Microscopy Data Bank (EMDB) under accession code EMD-44925, and model coordinates have been deposited in the Protein Data Bank (PDB) under accession ID 9BUY. All data needed to evaluate the conclusions in the paper are present in the paper and/or the Supplementary Materials.

Submitted 11 May 2024
Accepted 11 February 2025
Published 19 March 2025
10.1126/sciadv.adq3971
