# Supplementary material for: Structure and dynamics determine G protein coupling specificity at a class A GPCR
Source: Sci Adv. 2025 Mar 19;11(12):eadq3971. doi: 10.1126/sciadv.adq3971 (PMC12101717; doi:10.1126/sciadv.adq3971)
Supplement: Supplementary file 2 — Figs. S1 to S6 Tables S1 to S3 Legends for data S1 and S2 [file sciadv.adq3971_sm.v2.pdf]

ERRATUM PUBLISHED 27 JUNE 2025; SEE BELOW

Science Advances
AAAS

Supplementary Materials for

Structure and dynamics determine G protein coupling specificity at a class A GPCR

Marina Casiraghi *et al.*

Corresponding author: Peter Gmeiner, peter.gmeiner@fau.de; Michael T. Lerch, mlerch@mcw.edu; Brian K. Kobilka, kobilka@stanford.edu

Sci. Adv. **11**, eadq3971 (2025)
DOI: 10.1126/sciadv.adq3971

The PDF file includes:

Figs. S1 to S6
Tables S1 to S3
Legends for data S1 and S2

Other Supplementary Material for this manuscript includes the following:

Data S1 and S2

Erratum (27 June 2025): The original version of the Research Article, "Structure and dynamics determine G protein coupling specificity at a class A GPCR" by M. Casiraghi *et al*. contained an error in the calculation of the concentration of LM189 in the raw data. The files for data S1 and S2 have been corrected to reflect the updated raw data. In the Supplementary Materials PDF, instances of "Gs" were changed to "mini-Gs" and "Gi" to "mini-Gs/I" in the table shown in fig. S1D and its legend. The Supplementary Materials PDF and data S1 and S2 have been corrected.
Please see 10.1126/sciadv.adz4217 for additional corrections made to the main text.

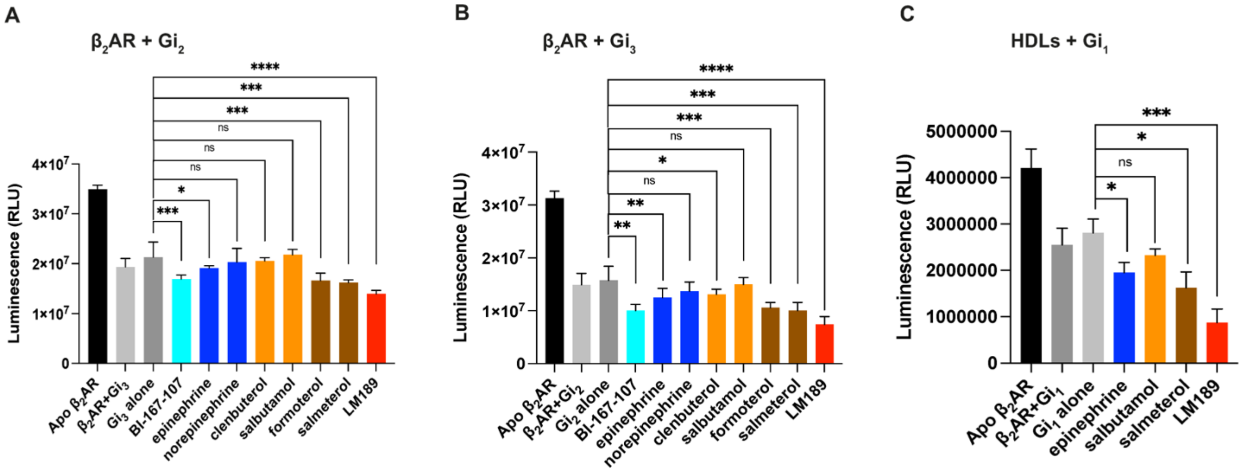

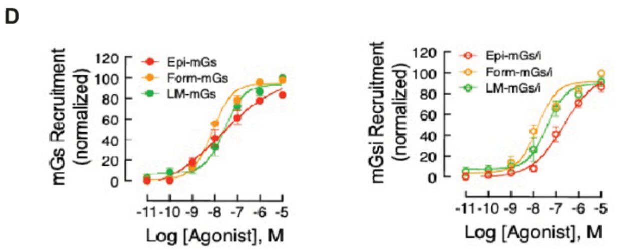

	mini-Gs		
Ligand	Log(t/Ka)	ΔLog(t/Ka)	RE
Epinephrine	7.9 ± 0.12	0	1
Formoterol	8.03 ± 0.11	0.13 ± 0.14	1.35*
LM189	7.55 ± 0.12	0.34 ± 0.14	0.45*

*=P<0.05

	mini-Gs/i		
Ligand	Log(t/Ka)	ΔLog(t/Ka)	RE
Epinephrine	6.89 ± 0.10	0	1
Formoterol	7.63 ± 0.13	0.74 ± 0.14	5.5*
LM189	7.5 ± 0.12	0.61 ± 0.14	4.07*

*=P<0.05

Ligand	ΔΔLog(t/Ka)	BF
Epinephrine	0	1
Formoterol	0.61 ± 0.2	4*
LM189	0.95 ± 0.2	9*

*=P<0.05

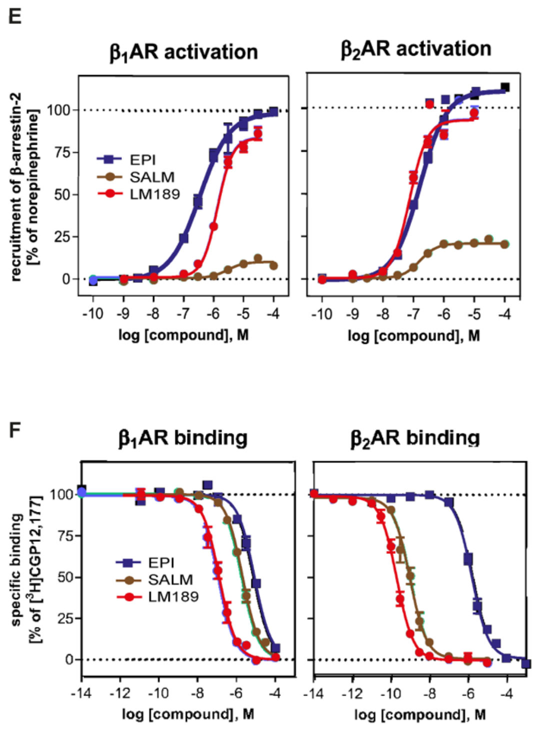

Fig. S1.

Characterization of the LM189 Gi-biased ligand. (A-B) Luminescence GTP-ase-Glo assays of β2AR coupled to (**A**) Gαi2 and (**B**) Gαi3 in the presence of ligands with different efficacies. (**A**) Salmeterol promotes higher Gαi2 turnover compared to the G-protein alone control (***P=0.0004). LM189 is more efficacious than salmeterol (****P≤0.0001). **(B)** Salmeterol promotes higher Gi3 turnover compared to the G-protein alone control (**P=0.0019). LM189 is more efficacious than salmeterol (****P<0.0001) **(C)** GTP-ase-Glo assay of β2AR reconstituted in HDLs particles and coupled to Gαi1 in the presence of ligands with different efficacies. LM189 promotes higher G-protein turnover compared to Gαi1 alone (***P=0.0003). In A-C, data are presented as the mean ± s.d. of biological triplicates. P values were calculated using the unpaired t test analysis on GraphPad Prism, assuming Gaussian distributions. Ns=(P>0.05), * (P≤0.05), ** (P≤0.01), *** (P≤0.001), **** (P≤0.0001). **(D)** LM189 bias quantification. Top panels: concentration-response curves of mini-Gs (left) and mini-Gs/i (right) activation mediated by epinephrine (red), formoterol (orange) and LM189 (green). Data points represent the mean ± s.d. of n=8 experiments. Upper and middle table: Δlog(τ/KA) ratios for mini-Gs (upper table) and mini-Gs/i (middle table) calculated from the log(τ/KA) ratios obtained from dose-response curves, considering epinephrine as the reference ligand. The relative effectiveness (RE)

of the ligands toward each pathway, relative to epinephrine, was determined. Lower table: ΔΔlog(τ/KA) ratios calculated from the Δlog(τ/KA) ratios. The ligand bias factors (BF), relative to epinephrine, were determined. Data were analyzed in a pairwise manner using a two-tailed unpaired student's t-test to determine the significance of the ligand biases, where $P<0.05$ was considered to be significant. **(E)** β-Arrestin-2 recruitment of epinephrine-bound and LM189-bound β_1- and β_2AR. Data are represented as the mean ± s.d. of 6-17 independent experiments each conducted in duplicates. **(F)** Competition binding experiments of LM189 at the β_1- and β_2-adrenoreceptor in comparison to the reference ligands epinephrine and salmeterol. Data are represented as the mean ± s.d. of 7-11 single experiments each performed in triplicates.

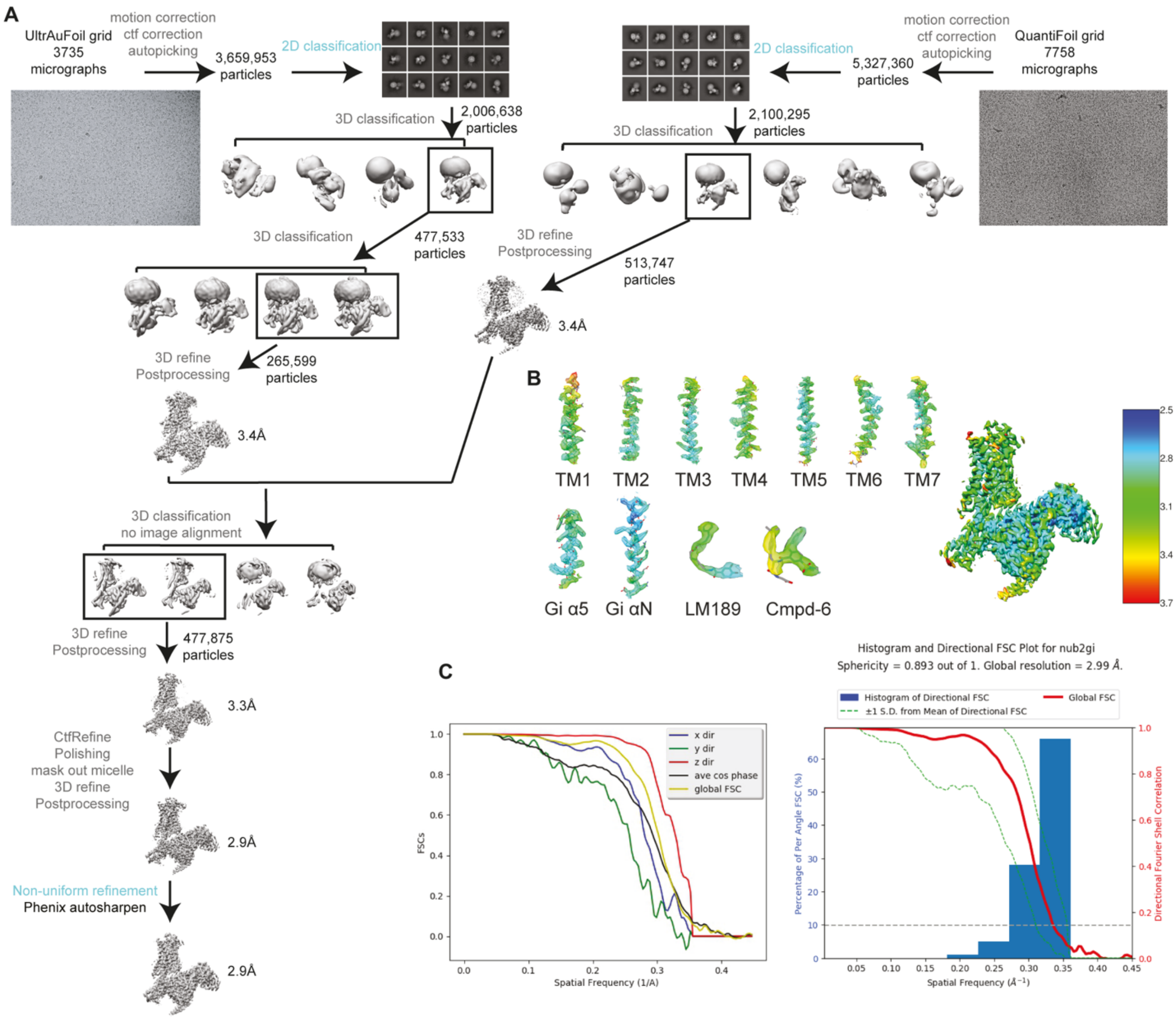

Fig. S2.

Cryo-EM data processing workflow and validation (A) cryo-EM data processing workflow. For detailed information please refer to Materials and Methods section. **(B)** Local resolution information of the final cryo-EM map. Local resolution colored EM densities and models are also shown for TM1-TM7, Gi α5, Gi αN, LM189, and Cmpd-6FA. Densities were visualized with UCSF ChimeraX (*68*). **(C)** Directional resolution validated by 3DFSC.

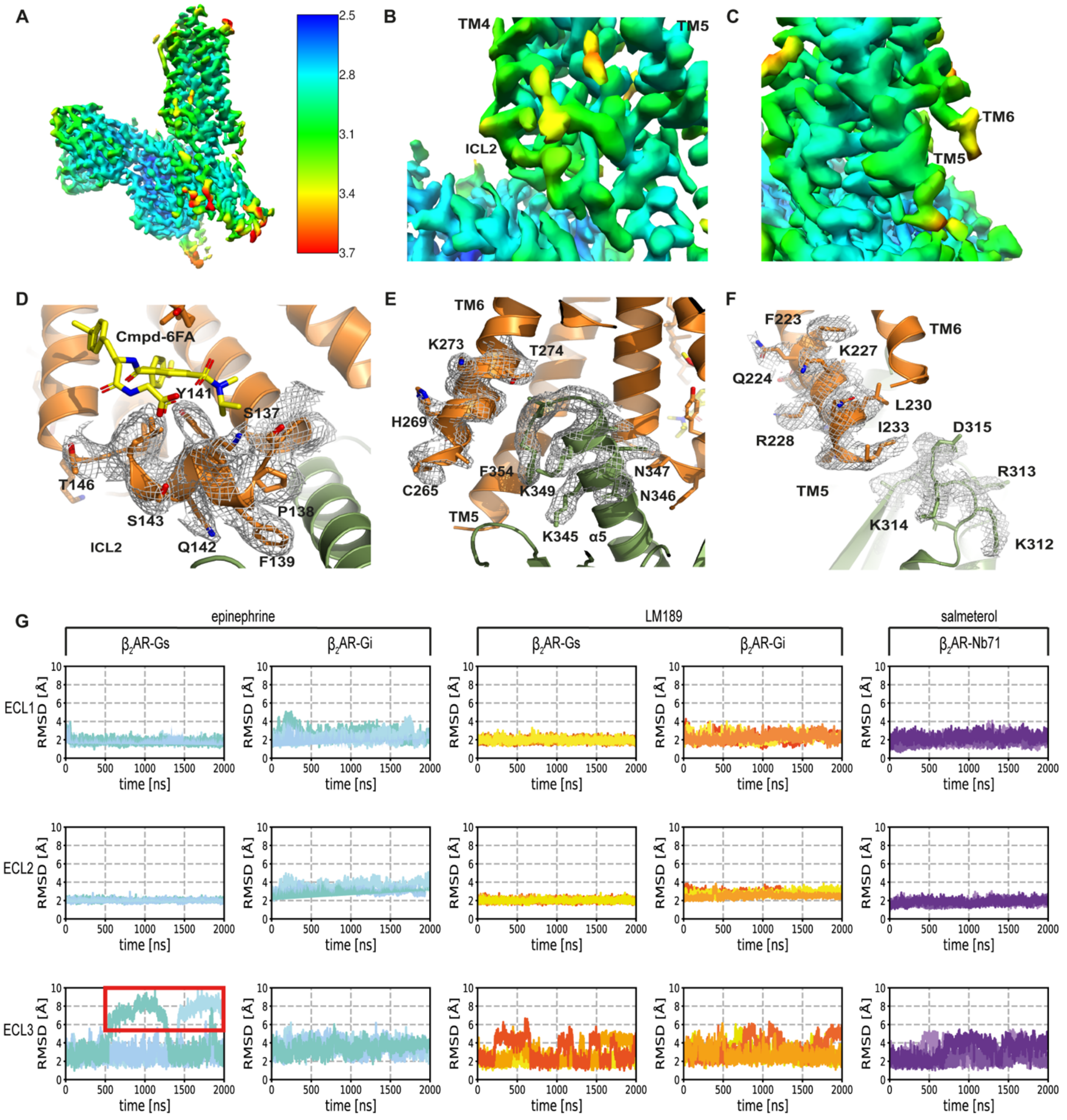

Fig. S3.

The LM189-bound β_2AR-Gi complex structure. (A-C) Local resolution Cryo-EM estimate maps. **(A)** Local resolution estimate map of the LM189-bound β_2AR-Gi complex. **(B)** Close-up of the local resolution estimate map around ICL2 of β_2AR. **(C)** Close-up of the local resolution estimate map around TM5 of β_2AR. **(D-F)** Cryo-EM densities of the β_2AR-Gi complex **(D)** Density around ICL2 of the β_2AR-Gi complex **(E)** Density at the tip of TM6 and the alpha5 of Gi. **(F)** Density around the tip of TM5 and the GTP-ase domain of Gi. **(G)** MD simulations of the extracellular region of ligand-bound β_2AR active state. From left to right, replicates of epinephrine-bound β_2AR-Gs and β_2AR-Gi simulations are showed in aquamarine, light-blue and turquoise, LM189-bound β_2AR-Gs and β_2AR-Gi replicates are colored in orange, red and yellow and salmeterol-bound β_2AR-Nb71 replicates are colored in violet and lilac.

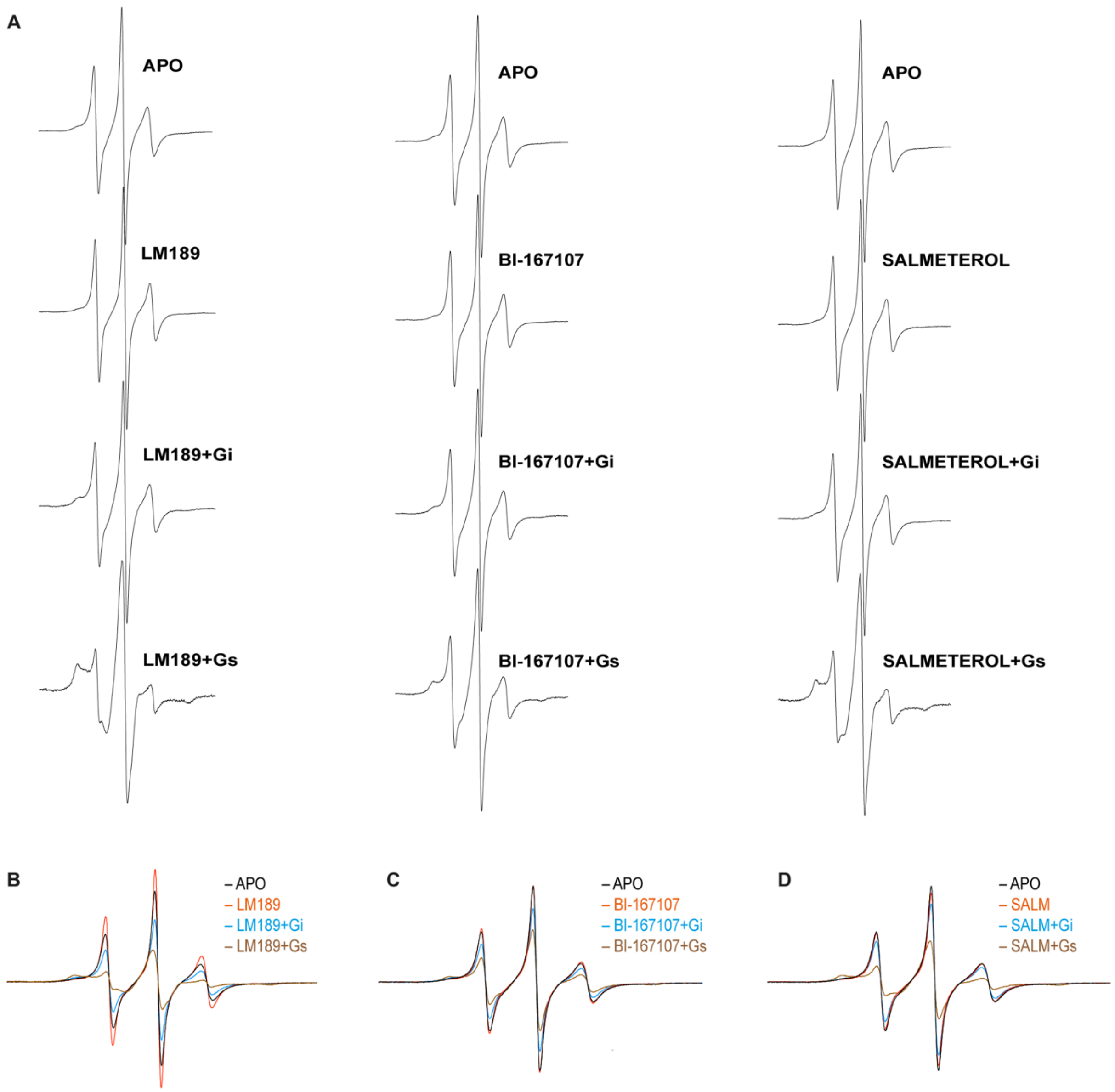

Fig. S4.

Cw-EPR studies of β$_2$AR ICL2 dynamics. (A) Amplitude normalized 142-IAP spectra for each of the experimental conditions tested. **(B-D)** Area-normalized 142-IAP spectra of the apo, ligand-bound, and G protein-bound receptor. Superimposed spectra are shown for the apo and **(B)** LM189, **(C)** BI-167107, and **(D)** salmeterol conditions.

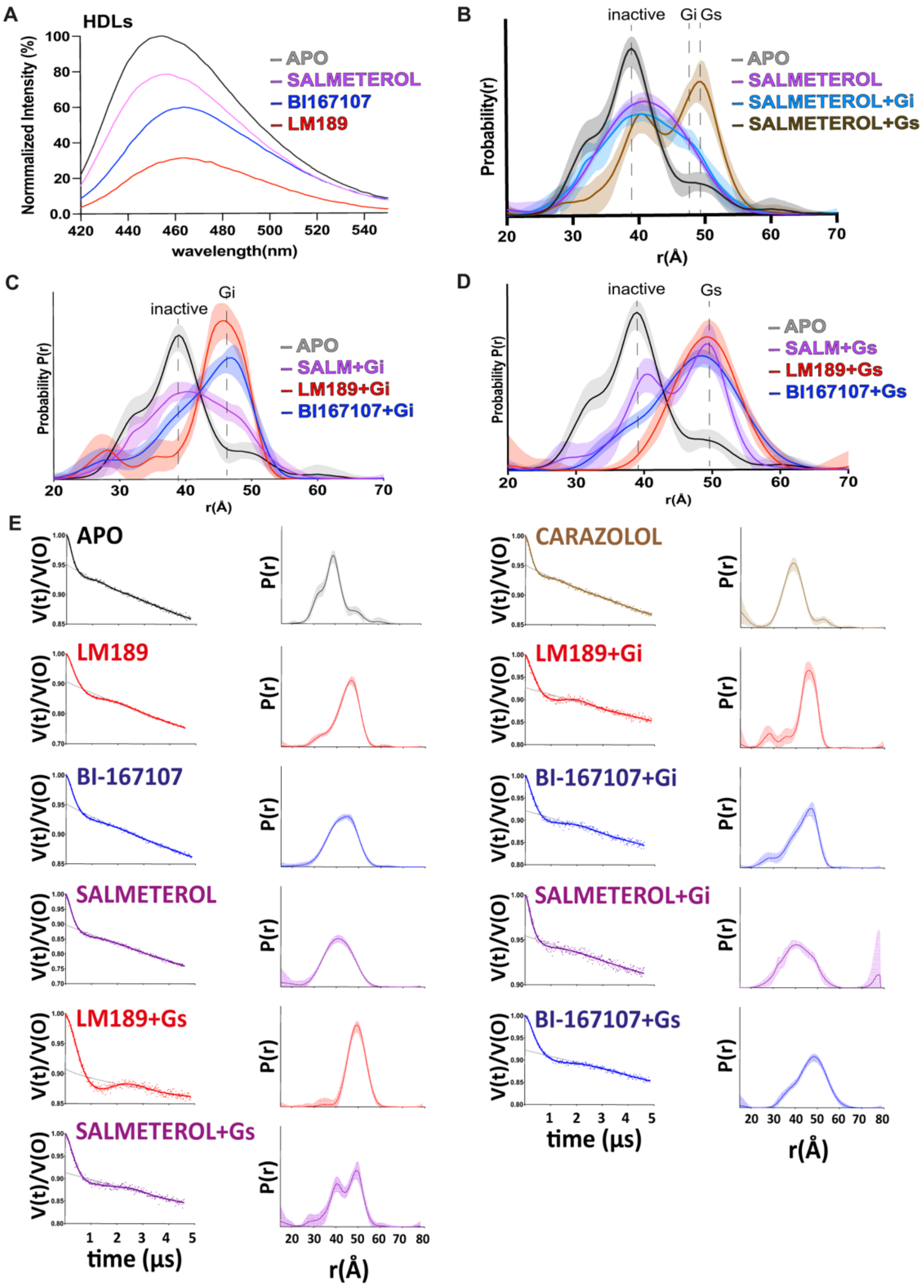

Fig. S5.

Investigation of TM6 conformational dynamics. (A) Steady-state fluorescence emission spectra of mBBr-labeled β2AR reconstituted in HDLs, in the presence of ligands. The spectra are normalized relative to apo (unliganded) receptor (gray). **(B-E)** DEER investigations of TM4/6 of β2AR in LMNG/CHS. Distance distributions in the presence of **(B)** salmeterol and G proteins, **(C)** Gi-bound receptor in the presence of different ligands, and **(D)** Gs-bound receptor in the presence of different ligands. **(E)** Background-subtracted dipolar evolution functions and distance distributions are shown for each sample. All data are color-coded as indicated.

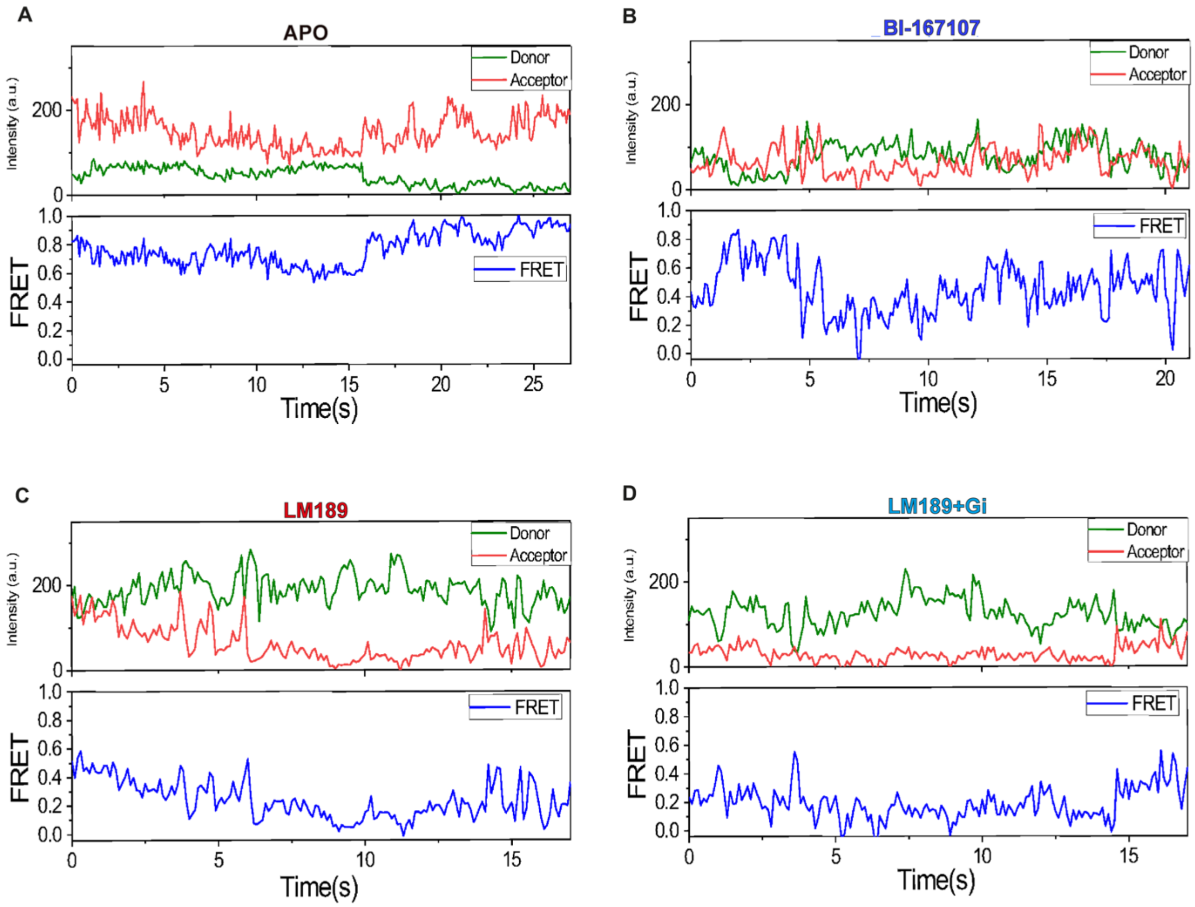

Fig. S6.

SmFRET representative traces. Donor and acceptor intensity values are reported in green and red respectively. Calculated FRET values are shown in blue. Example traces and FRET values are displayed for the apo condition **(A)**, for the full agonist BI-167107 **(B)**, for the Gi-biased ligand LM189 **(C)** and for LM189-bound receptor coupled to Gi and scfv-16 **(D)**.

Receptor Binding				
Compound	β_1AR		β_2AR	
	Ki [nM ± S.E.M][b]	n[c]	Ki [nM ± S.E.M][b]	n[c]
LM189	33 ± 5.6	7	0.058 ± 0.009	10
Epinephrine	2200 ± 290	8	380 ± 33	11
Salmeterol	520 ± 47	10	0.31 ± 0.064	9

β-arrestin 2 recruitment						
Compound	β_1AR			β_2AR		
	EC_{50}[nM ±S.E.M][e]	E_{max}[nM ±S.E.M][f]	n[g]	EC_{50}[nM ±S.E.M][e]	E_{max}[nM ±S.E.M][f]	n[g]
LM189	1300 ± 31	82 ± 3	6	71 ± 51	92 ± 3	6
Epinephrine	380 ± 52	100 ± 2	6	190 ± 6.9	111 ± 1	17
Salmeterol	3000 ± 820	10 ± 1	7	190 ± 16	21 ± 1	13

[a] Binding affinity determined in a radioligand displacement assay using membranes from HEK293T cells transiently transfected with the cDNA of β_1AR or β_2AR and the radioligand [^{3}H]CGP12,177. [b] Mean K_i values in [nM ± S.E.M.]. [c] Number of individual experiments each performed in triplicates. [d] Receptor mediated recruitment of β-arrestin-2 measured in HEK293T cells stably expressing the enzyme acceptor (EA) tagged β-arrestin-2 fusion protein and transiently transfected with the β_1AR or β_2AR each fused to the ProLink-PK1 fragment for enzyme complementation. [e] Potency displayed as mean EC_{50} value in [nM ± S.E.M.]. [f] Intrinsic activity E_{max} in [% ± S.E.M.] relative to the maximum effect of norepinephrine. [g] Number of individual experiments each performed in duplicates

Table S1.

Receptor binding data and functional data for β-arrestin-2 recruitment of LM189 at the β_1- and β_2-adrenoreceptor in comparison to the references epinephrine and salmeterol.

**Data collection and processing**	
Magnification	82,000
Voltage (kV)	300
Electron exposure	52.5
Defocus range (μm)	0.7-2
Pixel size (Å)	1.111
Symmetry imposed	C1
Initial particle images (no.)	8987313
Final particle images (no.)	477875
Map resolution (Å)	2.9
FCS threshold	2.5-3.7
Map resolution range (Å)	2.5-3.7

**Refinement**	
Initial model used (PDB code)	3SN6, 6DDE
Model resolution (Å)	3.2
FCS threshold	0.5
Model resolution range (Å)	3.2-28.9
Map sharpening *B* factor (Å^2)	-83.99
Model composition	
Non-hydrogen atoms	8640
Protein residues	1117
Ligands	5
B factors (Å^2)	2.5-3.7
Protein	63.61
Ligand	74.5
R.m.s. deviations	
Bond lenghts (Å)	0.003
Bond angles (°)	0.59
Validation	
MolProbity score	1.72
Clashscore	7.65
Poor rotamers (%)	0
Ramachandran plot	
Favored (%)	95.6
Allowed (%)	4.37
Disallowed (%)	0

Table S2.

Cryo-EM acquisition parameters of LM189-bound β_2AR-Gi$_1$-scFv16

Parameter	10W solid state amp	150W TWT amp
$\pi/2_{obs}$, π_{obs}, and π_{pump}	30.6 ns FWHM (72 ns time base)	15.3 ns FWHM (36 ns time base)
Δv (MHz)	45	90
d_1 (ns)	400	400
d_2 (ns)	3000-5000	3000-5000
Shot repetition time (μs)	4000	4000
Shots per point	10	4
Integration window (ns)	72	36

Table S3.
DEER data acquisition parameters.

Titles of excel tables in auxiliary materials:

Data S1: dose-response BRET
Data S2 : bias quantification
